# Supplementary material for: Maternal Immunization and Antenatal Care Situation Analysis (MIACSA) study protocol: a multiregional, cross-sectional analysis of maternal immunization delivery strategies to reduce maternal and neonatal morbidity and mortality
Source: BMJ Open. 2019 Jun 4;9(6):e024449. doi: 10.1136/bmjopen-2018-024449 (PMC6561463; doi:10.1136/bmjopen-2018-024449)
Supplement: Author's manuscript [file bmjopen-2018-024449.draft_revisions.pdf]

BMJ Open is committed to open peer review. As part of this commitment we make the peer review history of every article we publish publicly available.

When an article is published we post the peer reviewers' comments and the authors' responses online. We also post the versions of the paper that were used during peer review. These are the versions that the peer review comments apply to.

The versions of the paper that follow are the versions that were submitted during the peer review process. They are not the versions of record or the final published versions. They should not be cited or distributed as the published version of this manuscript.

BMJ Open is an open access journal and the full, final, typeset and author-corrected version of record of the manuscript is available on our site with no access controls, subscription charges or pay-per-view fees (http://bmjopen.bmj.com).

If you have any questions on BMJ Open's open peer review process please email info.bmjopen@bmj.com

BMJ Open

The Maternal Immunization and Antenatal Care Situation Analysis (MIACSA) study protocol: A multi-regional, cross-sectional analysis of maternal immunization delivery strategies to reduce maternal and neonatal morbidity and mortality

Journal:	*BMJ Open*
Manuscript ID	bmjopen-2018-024449
Article Type:	Protocol
Date Submitted by the Author:	26-May-2018

Complete List of Authors:	Roos, Nathalie; Organisation mondiale de la Sante, Department of Maternal Newborn Child and Adolescent Health (MCA), Epidemiology Monitoring and Evaluation (EME)<br>Lambach, Philipp; Organisation mondiale de la Sante, Department of Immunization, Vaccines and Biologicals (IVB), Initiative for Vaccine Research (IVR)<br>Mantel, Carsten; MMGH Consulting GmbH; Robert Koch Institute, Department of Infectious Disease Epidemiology<br>Mason, Elizabeth; London School of Hygiene and Tropical Medicine<br>Munoz, Flor M.; Baylor Coll Med, Department of Pediatrics, Section of Infectious Diseases<br>Giles, Michelle; Monash University and Monash Health, Department of Obstetrics and Gynaecology<br>MIACSA, expert advisory panel group<br>Moran, A; World Health Organization, Department of Maternal Newborn Child and Adolescent Health (MCA), Epidemiology Monitoring and Evaluation (EME)<br>Hombach, Joachim; Organisation mondiale de la Sante, Department of Immunization, Vaccines and Biologicals (IVB), Initiative for Vaccine Research (IVR)<br>Diaz, Theresa; World Health Organization, Department of Maternal Newborn Child and Adolescent Health (MCA), Epidemiology Monitoring and Evaluation (EME)

Keywords:	maternal immunization, maternal mortality, neonatal mortality, study protocol, maternal tetanus, neonatal tetanus

SCHOLARONE™
Manuscripts

The Maternal Immunization and Antenatal Care Situation Analysis (MIACSA) study protocol: A multi-regional, cross-sectional analysis of maternal immunization delivery strategies to reduce maternal and neonatal morbidity and mortality

Nathalie Roos[1]*, Philipp Lambach[2]*, Carsten Mantel[3], Elizabeth Mason[4], Flor M. Muñoz[5], Michelle L. Giles[6], MIACSA expert advisory panel group**, Allisyn C. Moran[1], Joachim Hombach[2]***, Theresa Diaz[1]***

1 Department of Maternal Newborn Child and Adolescent Health (MCA), Epidemiology Monitoring and Evaluation (EME), World Health Organization (WHO), Geneva, Switzerland

2 Department of Immunization, Vaccines and Biologicals (IVB), Initiative for Vaccine Research (IVR), WHO, Geneva, Switzerland

3 MMGH Consulting GmbH, Zürich, Switzerland and Department of Infectious Disease Epidemiology, Robert Koch Institute, Berlin, Germany

4 London School of Hygiene and Tropical Medicine, London, UK

5 Department of Pediatrics, Section of Infectious Diseases, Baylor College of Medicine, Houston, Texas, USA

6 Department of Obstetrics and Gynaecology, Monash University and Monash Health, Melbourne, VIC, Australia

* First authors

** The MIACSA expert advisory panel (EAP) consists of the following members: Flor M. Muñoz (chair), Michelle L. Giles (co-chair), Mercy Ahun, Martina Baye, Pradeep Haldar, Matthews Mathai, and Stephen Hodgins. Observers to EAP meetings included: Carsten

For peer review only

For peer review only - http://bmjopen.bmj.com/site/about/guidelines.xhtml

Mantel, Elizabeth Mason, Sonja Mertens, Jayani Pathirana, Sarah Rendell. Additional WHO experts included: Emily Wootton, Laura Nic Lochlainn, Ahmadu Yakubu, and Sara Rendell.

*** Last authors

Corresponding author: Dr Nathalie Roos, Department of Maternal Newborn Child and Adolescent Health (MCA), Epidemiology Monitoring and Evaluation (EME), WHO, Geneva, Switzerland, email: roosn@who.int, phone: +41 22 791 3191

Word count: 2,672

Key words: maternal immunization; maternal tetanus vaccination, maternal health; neonatal health; maternal tetanus; neonatal tetanus; maternal mortality; neonatal mortality; health care service delivery; antenatal care capacity; study protocol

For peer review only

ABSTRACT

Introduction. Maternal immunization (MI) is a safe and cost-effective way of preventing neonatal tetanus, and is one of several strategies that aim to reduce the high global rates of maternal and neonatal morbidity and mortality caused by vaccine-preventable diseases. Given the prospect of introducing new maternal vaccines in the near future, it is essential to identify and understand current policies, practices, and unmet needs for introducing and/or scaling up MI in low and middle income countries (LMICs).

Methods and analysis. The Maternal Immunization and Antenatal Care Situation Analysis (MIACSA) is a mixed methods, cross-sectional study that will collect data in four phases: (1) a review of global databases for selected health indicators in 136 LMICs; (2) a structured online survey directed at Maternal, Newborn, and Child Health (MNCH) and Expanded Programme on Immunization (EPI) focal points in all 136 LMICs; (3) semi-structured telephone interviews of 30 selected LMICs; and (4) 10 week-long country visits, including key informant interviews, health facility visits, and focus group discussions. The principal analyses will assess correlations between the various aspects of MI delivery strategies and proxy measures of health systems performance related to vaccine-preventable disease control. The primary outcome will be a typology of existing MI delivery models, and secondary outcomes will include country profiles of child and maternal health indicators, and an MI gaps and needs analysis.

Ethics and dissemination. The protocol was approved by the World Health Organization Ethics Review Committee. The results will be made available in a project report and submitted for publication in peer-reviewed journals that will be shared broadly among global health decision makers, researchers, product developers, and country-level stakeholders.

Registration. Not applicable.

For peer review only - http://bmjopen.bmj.com/site/about/guidelines.xhtml

For peer review only

For peer review only - http://bmjopen.bmj.com/site/about/guidelines.xhtml

ARTICLE SUMMARY

Strengths and limitations of this study

- The MIACSA study will provide a global overview and analysis of existing maternal immunization (MI) delivery strategies in low- and middle-income countries (LMICs).
- In order to optimise the assessment of MI delivery strategies in LMICs, data will be collected in four phases: (1) a desktop review of relevant health indicators from global sources, e.g. WHO and other UN databases, from 136 LMICs; (2) a structured online survey directed at Maternal, Neonatal, and Child Health (MNCH) and Expanded Programme on Immunization (EPI) programme managers and focal points in all 136 LMICs; (3) semi-structured telephone interviews of 30 selected LMICs; and (4) 10 week-long country visits, including key informant interviews, health facility visits, and focus group discussions.
- Strengths of the study include a mixed-methods design; a multidisciplinary approach leveraging policy-level, academic, and implementers' experience; multi-level data collection; a globally comprehensive analysis combined with in-depth information of a subsample of LMICs; inter-sectoral collaboration between MNCH and EPI programmes, and broad dissemination of results.
- The results will provide evidence for a typology of MI delivery models in LMICs, and identify capacity needs and key system changes, including policy adjustments required to introduce new maternal vaccines and/or scale up existing MI in LMICs.
- Limitations include the small number of countries and health care facilities visited within each country included in the study, thus precluding generalization of country visit findings to a national level, and the fact that the end-users' perspective will be captured only indirectly through community health workers.

For peer review only - http://bmjopen.bmj.com/site/about/guidelines.xhtml

For peer review only

For peer review only - http://bmjopen.bmj.com/site/about/guidelines.xhtml

INTRODUCTION

Vaccine-preventable diseases are a major cause of global child morbidity and mortality, particularly in low- and middle-income countries (LMICs).[1] Since the 1990s, public health interventions have more than halved under-five childhood mortality; however, reduction of stillbirths and of neonatal mortality (death in the first 28 days of life) has been slower.[2] This is in part due to the fact that most vaccines cannot be administered to newborns, who, being unable to develop protective responses due to limitations in their immune system, are left particularly vulnerable to infectious diseases. Vaccination of pregnant women, or maternal immunization (MI), has proven to be an effective strategy to reduce neonatal tetanus, and is a potential strategy to reduce the burden of other vaccine-preventable diseases in mothers and infants. Thus, MI is one of several strategies that aim to reach the third sustainable development goal of ending preventable maternal and newborn deaths.[3-5]

Studies have shown that MI can effectively protect the mother, as well as her child, through transplacental transfer of maternal immunoglobulin G (IgG) to the foetus.[6, 7] The Maternal and Neonatal Tetanus Elimination (MNTE) initiative has led the way in the implementation of MI, combining at least two doses of tetanus toxoid containing vaccine (TTCV) during pregnancy (TT2+) with the promotion of hygienic delivery and clean cord care practices, as well as vaccination of children and women of reproductive age, to eliminate maternal and neonatal tetanus as a public health problem. Between the late 1980s and 2015, the MNTE initiative reduced global tetanus-related neonatal mortality by 96%.[8]

TTCV and inactivated influenza vaccines are considered safe and effective for use during pregnancy,[9] and are recommended for pregnant women by the World Health Organization

For peer review only - http://bmjopen.bmj.com/site/about/guidelines.xhtml

(WHO).[6, 10-13] New vaccines, several of which are under development and evaluation, target other important pathogens, such as group B streptococcus (GBS), and respiratory syncytial virus (RSV), and may provide safe and cost-effective protection of mothers and their infants through MI in the future.[14-18]

In order to identify the challenges of implementing current and new vaccines for MI, a better understanding is needed of the capabilities and limitations of existing delivery platforms, such as antenatal care (ANC) services and the Expanded Programme on Immunization (EPI).[19] The capacity of ANC services to deliver vaccines to pregnant women will require thorough assessment, as globally only 62% of women benefit from at least four ANC visits, i.e. the proportion of pregnant women who received 4 or more ANC visits during their last pregnancy (ANC4+), and in Sub-Saharan Africa and South Asia, ANC4+ coverage is only 52% and 46%, respectively.[20] Delivering vaccinations and other essential interventions to women at the necessary timely intervals during pregnancy, as well as documenting the coverage and outcomes of such interventions, requires a robust ANC platform with sufficient personnel and resources.[21]

WHO recommends that pregnant women living in endemic areas are sufficiently immunised against tetanus in order to protect the women and their newborn infants. MI with TTCV is routine in many countries;[22, 23] however, progress of tetanus vaccination in LMICs has faced challenges leading to delays in elimination, and uptake among pregnant women of other vaccines, such as influenza and pertussis vaccines, has been low. As a part of EPI services, routine tetanus immunization during pregnancy has been complemented with supplementary vaccination activities in a majority of countries in order to reach high coverage and achieve

For peer review only - http://bmjopen.bmj.com/site/about/guidelines.xhtml

MNTE goals. A better understanding of MI in the context of both ANC and EPI, including implementation of guidelines and policies, ministerial responsibilities at national and subnational levels, vaccine management including cold chain and logistics, vaccine administration, staff capacity, social mobilisation, vaccine acceptance, and assessment of vaccine safety, may help identify service delivery challenges as well as opportunities to optimise current and future MI efforts.[24]

Closer collaboration between ANC and EPI services could provide a unique and cost-effective opportunity to further strengthen preventive health care measures for women and children under each programme, by reducing missed opportunities for immunization, including MI, as well as reinforcing the delivery of essential health care services.

In view of recent product and policy developments, the WHO, supported by the Bill and Melinda Gates Foundation, aims to identify the knowledge gaps in MI delivery strategies by mapping the strengths and challenges of existing ANC and immunization services for pregnant women in LMICs through the Maternal Immunization and Antenatal Care Situation Analysis (MIACSA) project. The results will provide the evidence for a typology of MI delivery models, as well as identify the capacity needs and key system changes required to introduce new maternal vaccines and/or strengthen vaccine delivery for MI in LMICs. Ultimately, the project aims to determine how existing health care services can be further strengthened to improve maternal and neonatal outcomes, and how they could accommodate new MI vaccines.

For peer review only

For peer review only - http://bmjopen.bmj.com/site/about/guidelines.xhtml

METHODS AND ANALYSIS

Study design and data collection.

Between November 2016 and December 2018, a mixed-methods, cross-sectional study will be carried out in four phases to assess key health system features related to the implementation of MI (Figure 1). An expert advisory panel (EAP) consisting of specialists in immunization, maternal and neonatal health, MI implementation and social sciences, will provide technical advice on the study design, the development of research questions and surveys, the data collection methods, and the results interpretation. In addition to following WHO standards for global monitoring surveys, all data collection tools and standard operating procedures will be reviewed and endorsed by the EAP. The surveys and country visits will be conducted in local languages when needed.

Data collection phase 1. The first phase will consist of collecting key health indicators of LMICs to create outlines of country profiles, focusing on ANC and EPI services. A desktop review of pre-defined health indicators (Figure 2) from 136 LMICs will be conducted from existing global data sources, including Demographic and Health Surveys (DHS)/Multiple Indicator Cluster Surveys (MICS), WHO/United Nations Children's Fund (UNICEF) estimates of national immunization coverage, WHO/UNICEF Joint Reporting Forms (JRF), MNTE reports, and WHO Maternal, Newborn, Child, and Adolescent Health (MNCAH) policy survey. The indicators will focus on governance and policy environment, health systems performance, and immunization activities, including MI. Data from phase 1 will be compiled in a database for analysis of the study's research questions, and will inform the selection of countries for phase 3 (see below).

For peer review only - http://bmjopen.bmj.com/site/about/guidelines.xhtml

Data collection phase 2. The country profiles established in phase 1 will be completed and, if needed, updated by an online survey with WHO Regional Offices, national Ministry of Health (MoH) focal points from Maternal, Newborn and Child Health (MNCH) and EPI programmes and their WHO Country Office counterparts in all LMICs, using a structured questionnaire (Figure 3). Data will be collected on service delivery models of maternal tetanus vaccination, including delivery platforms, programme funding, disease surveillance, and vaccine safety surveillance. Data on maternal vaccines other than tetanus will be included when pertinent. A draft questionnaire will be piloted in advance of the survey. Non-responders will be followed-up by telephone and email. Revisions following queries on missing, erroneous, or inconsistent data will be done at country level.

Data collection phase 3. In order to understand how existing health care delivery services could be adapted to implement MI beyond tetanus immunization, further data will be collected on delivery platforms for maternal tetanus vaccination in LMICs. In-depth telephone interviews will be conducted with EPI and MNCH programme officers responsible for MI at the MoH in a sample of 30 countries, using a semi-structured questionnaire (Figure 4). The countries will be selected based on performance of MI as assessed by coverage of maternal TTCV and ANC, geographic representation, and recommendations from WHO Regional Offices on MI priorities. The countries will be stratified into four groups; high and low maternal tetanus vaccination performance measured as protection at birth (PAB), i.e. the proportion of newborns protected at birth against neonatal tetanus, with a cut-off of 90%, and high and low ANC performance (with a cut-off of the median ANC4+ coverage in countries with available data). PAB was identified as a more reliable proxy measure than TT2+, as the issue of not including already vaccinated women in the numerator used for estimating the latter indicator would be avoided. The PAB cut-off level was set based on the target required

For peer review only - http://bmjopen.bmj.com/site/about/guidelines.xhtml

to attain and sustain MNTE, whereby >80% of pregnant women are immunized against tetanus. The country selection will include a representation of all MI delivery models and WHO regions, with a focus on Africa and South-East Asia where maternal and neonatal mortality are highest, and will ensure inclusion of high performing countries in order to include likely early adopters of new maternal vaccines and learning cases of best practices.

The interviews will collect data on the policy, governance, and funding environment for EPI and ANC programmes, ANC delivery, and maternal tetanus vaccination including monitoring and evaluation of results. The questionnaire will be shared with WHO country office focal points and MoH MNCH and EPI managers for compilation in advance of the teleconference, allowing for discussion and clarification when needed during the actual interview. Responses will be recorded using standard data entry procedures, and may be voice-recorded if consent is obtained by the interviewees. Any discordant responses will be attempted to be resolved by consensus, and incomplete responses will be followed up. A summary of key findings will be shared with the participants to confirm the responses were correctly captured.

Data collection phase 4. Finally, in-country visits will be conducted in order to collect data on MI from key decision-makers and implementers at every level of the health care system, as well as to determine actual delivery, capacity and coordination of ANC and EPI services, on both supply and demand sides of the health care services (Figure 5). Ten countries will be selected based on high, medium, or low performance of MI systems as assessed by PAB and TT2+, a range of different MI delivery models (e.g. degree of coordination between EPI and ANC in MI delivery), and agreement by senior national and subnational MNCH and EPI staff for study visits. The final country selection will ensure representation of the range of MI

For peer review only - http://bmjopen.bmj.com/site/about/guidelines.xhtml

delivery models, and will include high-performing countries, MNTE priority countries, and countries with high ANC4+ coverage. Site visits will include ANC and EPI sites and session observations, focus group discussions, and in-depth interviews. The week-long visits will be piloted in two countries to adjust and refine the data collection tools and the standard operating procedures, and data from these two countries will be included in the final analysis.

An initial joint focus group discussion will be held with national-level stakeholders, followed by key informant interviews with stakeholders pertinent to MI, ANC, and EPI services at subnational levels of the health care system, including decision and policy makers, technical and financial parties, and civil society, such as non-governmental organisations. The study will aim to conduct a total of twelve health facility visits taking into account a balance of geographical locations, urban and rural areas, and - if possible - different types of health facilities (e.g. small and larger health units). The country visits will be concluded with an on-site debriefing and joint data analysis with MoH MNCH and EPI focal points and other main country-level stakeholders. End-users, i.e. pregnant women, will not be interviewed as it would require a separate study design; however, their perspective will be indirectly included through the participation of community health workers at stakeholder meetings.

Data analysis plan.

The principal analyses will assess correlations between the various documented aspects of MI delivery platforms and metrics of health systems performance, i.e. PAB, ANC4+, and TT2+. The primary outcome will be a typology of MI delivery models with indicators of high performance and capacity to introduce new maternal vaccines and/or scale-up MI, based on the analysis of quantitative data collected during the four project phases. Secondary outcomes

For peer review only

For peer review only - http://bmjopen.bmj.com/site/about/guidelines.xhtml

will include country profiles of child and maternal health indicators with a special focus on MI and an MI gaps and needs analysis, based on intra- and cross-country analyses of qualitative data, according to themes generated from research questions and sub-themes generated from grounded analysis of data collected. The analyses will, where possible, take into consideration within-country heterogeneity, such as differences between urban and rural settings, and between various health system levels.

Furthermore, the data analyses will take into account the limitations of the study, including the reliability of the selected outcome measures, i.e. PAB and TT2+, potential biases introduced by the limited number of countries for which in-depth information will be available, i.e. through telephone interviews and in-country visits, selective sampling of in-country site visit locations, missing data, and the fact that the end-users' perspective will be captured only indirectly through community health workers.

For peer review only

ETHICS AND DISSEMINATION

Ethical considerations. The first three phases of the study are exempt from ethical permission as participants will provide information on operations and administration of public health services on a purely professional basis, and without disclosure of person-identifiable information. The protocol for the country visits was approved by the WHO Research Ethics Review Committee (ERC.0002908).

Country ownership will be ensured through transfer of responsibility for the data provision to in-country focal points, and by joint, on-site analysis of the data collected during the country visits with the main stakeholders. The study aims to contribute to the evidence needed to ensure more equitable access to high-impact global health interventions, such as MI.[25]

Data management and dissemination.

The data will be managed and analysed by data clerks who were not part of the data collection. Anonymised data from surveys and key informant interviews, excluding any confidential information as identified by the in-country focal points, will be uploaded to a publicly available data repository hosted by the WHO. Recordings from country interviews will be transcribed before the qualitative analyses and destroyed at completion of the data analyses.

The results will be submitted for publication in peer-reviewed journals, as well as in a MIACSA project report that will be shared widely with global health decision makers, researchers, product developers, and implementers. The report and/or specific aspects of the

For peer review only - http://bmjopen.bmj.com/site/about/guidelines.xhtml
For peer review only

project, will be presented at international stakeholder meetings, with the ultimate aim to establish a knowledge network of countries exploring MI implementation strategies. Further, the results will be shared through summaries on the WHO website and in public fora. Dissemination of the MIACSA results will aim to provide advice on best practices, policy requirements, capacity needs, and health system changes needed for successful introduction and integration of new maternal vaccines into national health systems, including ANC and EPI services, in LMICs.

For peer review only

For peer review only - http://bmjopen.bmj.com/site/about/guidelines.xhtml

REFERENCES

1. UNICEF WHO, and World Bank Group. Levels and Trends in Child Mortality: Report 2017. New York, 2017.

2. Lawn JE, Blencowe H, Oza S, et al. Every Newborn: progress, priorities, and potential beyond survival. *Lancet* 2014;384:189-205.

3. Sobanjo-Ter Meulen A, Abramson J, Mason E, et al. Path to impact: A report from the Bill and Melinda Gates Foundation convening on maternal immunization in resource-limited settings; Berlin - January 29-30, 2015. *Vaccine* 2015;33:6388-95.

4. You D, Hug L, Ejdemyr S, et al. Global, regional, and national levels and trends in under-5 mortality between 1990 and 2015, with scenario-based projections to 2030: a systematic analysis by the UN Inter-agency Group for Child Mortality Estimation. *Lancet* 2015;386:2275-86.

5. WHO. Trends in maternal mortality: 1990 to 2015. Estimates by WHO, UNICEF, UNFPA, World Bank Group and the United Nations Population Division. Geneva: World Health Organization, 2015.

6. Englund JA, Mbawuike IN, Hammill H, et al. Maternal immunization with influenza or tetanus toxoid vaccine for passive antibody protection in young infants. *J Infect Dis* 1993;168:647-56.

7. Puck JM, Glezen WP, Frank AL, et al. Protection of infants from infection with influenza A virus by transplacentally acquired antibody. *J Infect Dis* 1980;142:844-9.

8. WHO. http://www.who.int/immunization/diseases/MNTE_initiative/en/ Accessed 22 January 2018.

9. Keller-Stanislawski B, Englund JA, Kang G, et al. Safety of immunization during pregnancy: a review of the evidence of selected inactivated and live attenuated vaccines. *Vaccine* 2014;32:7057-64.

For peer review only - http://bmjopen.bmj.com/site/about/guidelines.xhtml

10. WHO. Safety of immunization during pregnancy: A review of the evidence. World Health Organization. Geneva, 2014.

11. Hviid A, Svanstrom H, Molgaard-Nielsen D, et al. Association Between Pandemic Influenza A(H1N1) Vaccination in Pregnancy and Early Childhood Morbidity in Offspring. *JAMA Pediatr* 2017;171:239-48.

12. Vaccines against influenza WHO position paper - November 2012. *Wkly Epidemiol Rec* 2012;87:461-76.

13. Eick AA, Uyeki TM, Klimov A, et al. Maternal influenza vaccination and effect on influenza virus infection in young infants. *Arch Pediatr Adolesc Med* 2011;165:104-11.

14. Russell LB, Kim SY, Cosgriff B, et al. Cost-effectiveness of maternal GBS immunization in low-income sub-Saharan Africa. *Vaccine* 2017;35:6905-14.

15. Donders GG, Halperin SA, Devlieger R, et al. Maternal Immunization With an Investigational Trivalent Group B Streptococcal Vaccine: A Randomized Controlled Trial. *Obstet Gynecol* 2016;127:213-21.

16. Chaithongwongwatthana S, Yamasmit W, Limpongsanurak S, et al. Pneumococcal vaccination during pregnancy for preventing infant infection. *Cochrane Database Syst Rev* 2015;1:CD004903.

17. PATH. Advancing maternal immunization (AMI) http://sites.path.org/cvia/our-disease-targets/respiratory-syncytial-virus/advancing-maternal-immunization-ami/2017 Accessed 22 January 2018.

18. Glenn GM, Smith G, Fries L, et al. Safety and immunogenicity of a Sf9 insect cell-derived respiratory syncytial virus fusion protein nanoparticle vaccine. *Vaccine* 2013;31:524-32.

For peer review only

19. Singh K, Story WT, Moran AC. Assessing the Continuum of Care Pathway for Maternal Health in South Asia and Sub-Saharan Africa. *Matern Child Health J* 2016;20:281-9.

20. UNICEF. Antenatal care coverage: At least four visits https://data.unicef.org/topic/maternalhealth/antenatal-care/#2017 Accessed 10 April 2017.

21. Hodgins S, D'Agostino A. The quality-coverage gap in antenatal care: toward better measurement of effective coverage. *Glob Health Sci Pract* 2014;2:173-81.

22. Pecenka C, Munthali S, Chunga P, et al. Maternal influenza immunization in Malawi: Piloting a maternal influenza immunization program costing tool by examining a prospective program. *PLoS One* 2017;12:e0190006.

23. McMorrow ML, Emukule GO, Obor D, et al. Maternal influenza vaccine strategies in Kenya: Which approach would have the greatest impact on disease burden in pregnant women and young infants? *PLoS One* 2017;12:e0189623.

24. Nesin M, Read J, Koso-Thomas M, et al. Maternal Immunization: Current status and future prospects. *Vaccine* 2015;33:6371.

25. Verweij M, Lambach P, Ortiz JR, et al. Maternal immunisation: ethical issues. *Lancet Infect Dis* 2016;16:e310-e14.

For peer review only - http://bmjopen.bmj.com/site/about/guidelines.xhtml

AUTHORS' CONTRIBUTIONS

NR and PL designed the study; CM, EM, FMM, MLG, and the MIACSA expert advisory panel group drafted the protocol with NR and PL; and all authors reviewed and approved the final manuscript version.

ACKNOWLEDGMENTS

The investigators wish to thank the EPI and MNCH focal points at the regional and national WHO offices for their helpful contributions to the planning of the study, and the external specialists of the Expert Advisory Panel for their valuable advice and guidance on development of the protocol methodology. The authors also wish to thank Dr. Peter Mark Jourdan for drafting and editing the manuscript.

FUNDING

This work was supported by the Bill and Melinda Gates Foundation, grant number OPP1156378.

COMPETING INTERESTS

The authors declare no competing interests.

For peer review only

For peer review only - http://bmjopen.bmj.com/site/about/guidelines.xhtml

FIGURE LEGENDS

Figure 1. Key health system features studied by the MIACSA project.

EPI = Expanded Programme on Immunization, ANC = antenatal care, AEFI = adverse events following immunization, TT = tetanus toxoid.

Figure 2. Study phase 1: List of indicators for the review of global databases.

CES = coverage evaluation survey, WUENIC = World Health Organization (WHO)/United Nations Children's Fund (UNICEF) estimates of national immunization coverage, BCG = Bacillus Calmette-Guérin vaccine, DPT1 = first dose of diphtheria-pertussis-tetanus vaccine, DPT3 = third dose of diphtheria-pertussis-tetanus vaccine, HepB1 = first dose of hepatitis B vaccine, HepB3 = third dose of hepatitis B vaccine, Hib1 = first dose of *Haemophilus influenzae* type B vaccine, Hib3 = third dose of *H. influenzae* type B vaccine, MCV1 = first dose of measles-containing vaccine, MCV2 = second dose of measles-containing vaccine, PcV1 = first dose of pneumococcal conjugate vaccine, PcV3 = third dose of pneumococcal conjugate vaccine, Pol1 = first dose of polio-containing vaccine, Pol3 = third dose of polio-containing vaccine, TT1 = first dose of tetanus toxoid vaccine, TT1+ = at least one dose of tetanus toxoid vaccine, TT2 = second dose of tetanus toxoid vaccine, TT2+ = at least two doses of tetanus toxoid vaccine, TT3 = third dose of tetanus toxoid vaccine, TT4 = fourth dose of tetanus toxoid vaccine, TT5 = fifth dose of tetanus toxoid vaccine, RCV1 = first dose of rubella-containing vaccine, RotaC = second or third dose of rotavirus vaccine depending on number of doses recommended in national schedule, YFV = yellow fever vaccine.

Figure 3. Study phase 2: Variables collected from online survey of 136 LMICs.

For peer review only - http://bmjopen.bmj.com/site/about/guidelines.xhtml

373 A structured questionnaire will be used to determine which service delivery platforms are in
374 place for tetanus vaccination of pregnant women in low- and middle-income countries
375 (LMICs), and to understand how existing health services could be adapted to implement
376 maternal immunization beyond tetanus vaccination. Internal validation questions are
377 incorporated in the questionnaire, and sources of data are requested, i.e. if administrative data
378 or personal estimates. ANC = antenatal care, EPI = Expanded Programme on Immunization,
379 TT = tetanus toxoid, Td = tetanus-diphtheria, Tdap = tetanus-diphtheria-acellular pertussis,
380 AEFI = adverse events following immunization, TT2+ = at least 2 doses of tetanus toxoid
381 vaccine during pregnancy, PAB = protection at birth, BCG = Bacillus Calmette-Guérin
382 vaccine, OPV = oral polio vaccine, HBV = hepatitis B vaccine.

383

384 **Figure 4. Study phase 3: Variables collected from interviews of 30 selected LMICs.**

385 A semi-structured questionnaire will be used to assess the preparedness of antenatal care
386 services for introducing (additional) immunizations for pregnant women in selected low- and
387 lower-middle income countries, and to understand the strengths and weaknesses of current
388 immunization to guide future planning. Internal validation questions are incorporated and
389 probing for further details will be done when deemed necessary by the interviewer(s).
390 Sources of data provided are requested, i.e. if administrative data or personal estimates. ANC
391 = antenatal care, EPI = Expanded Programme on Immunization, NITAG = National
392 Immunisation Technical Advisory Group, HMIS = Health management Information System,
393 TT = tetanus toxoid, Td = tetanus-diphtheria, AEFI = adverse events following
394 immunization.

395

396 **Figure 5. Study phase 4: Country study analysis framework for 10 country visits.**

For peer review only - http://bmjopen.bmj.com/site/about/guidelines.xhtml

397 Key informant interviews, health facility visits, and focus group discussions will enable observation and collection of further data on the variables from the previous study phases, in particular at different levels of the health care system, and of socio-cultural and socio-economic factors. End-users, i.e. pregnant women, will not be interviewed as it would require a separate study design, and their perspective will be indirectly included through the participation of community health workers at stakeholder meetings.

For peer review only

For peer review only - http://bmjopen.bmj.com/site/about/guidelines.xhtml

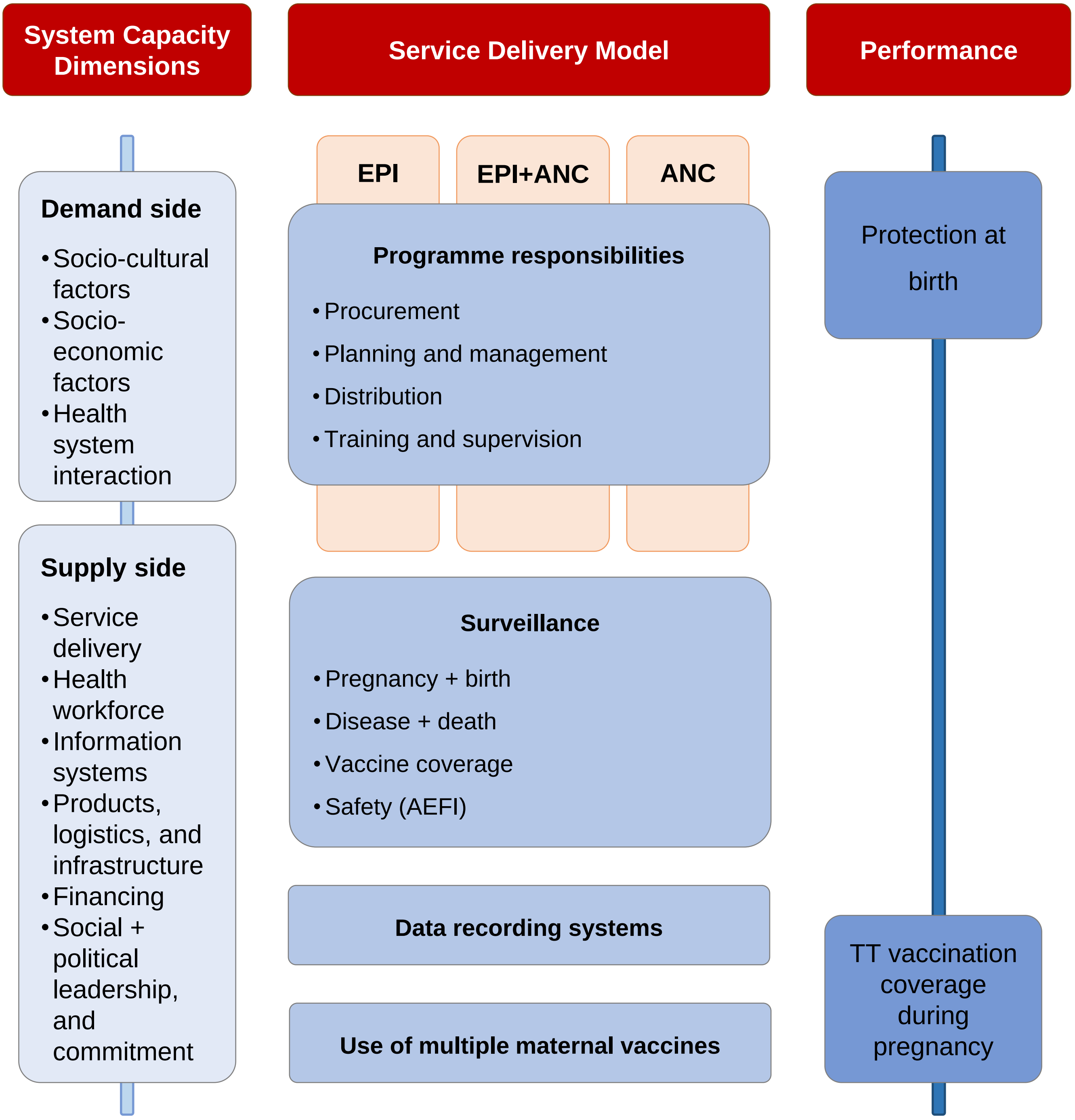
System Capacity Dimensions
Service Delivery Model
Performance
Demand side
•Socio-cultural factors
•Socio-economic factors
•Health system interaction
Supply side
•Service delivery
•Health workforce
•Information systems
•Products, logistics, and infrastructure
•Financing
•Social + political leadership, and commitment
EPI
EPI+ANC
ANC
Programme responsibilities
•Procurement
•Planning and management
•Distribution
•Training and supervision
Surveillance
•Pregnancy + birth
•Disease + death
•Vaccine coverage
•Safety (AEFI)
Data recording systems
Use of multiple maternal vaccines
Protection at birth
TT vaccination coverage during pregnancy

General

- World Bank income classification;
- Female literacy rate.

Health systems

General

- Health systems classification;
- Birth cohort (most recent year of available data);
- Target population of pregnant women.

Governance and policy environment

- Percentage of total expenditure on routine immunization financed by government funds;
- Existence of national immunization technical advisory group (NITAG);
- National policy on minimum antenatal care (ANC) visits;
- Eligibility for global vaccine alliance (GAVI) support.

Health systems performance

- Maternal, neonatal, and infant mortality;
- Stillbirth rates;
- Physician and midwife densities;
- Institutional deliveries;
- Coverage of a minimum of four ANC visits (ANC4+).

Vaccination, including maternal immunization

- Number of confirmed tetanus and neonatal cases;
- Coverage of at least 2 doses of TT vaccine during pregnancy (TT2+);
- Tetanus toxoid (TT) vaccine as a proportion of CES and WUENIC vaccines, i.e. BCG, DPT1, DPT3, HepB1, HepB3, Hib1, Hib3, MCV1, MCV2, PAB, PcV1, PcV3, Pol1, Pol3, TT1, TT1+, TT2, TT2+, TT3 TT4, TT5, RCV1, RotaC, YFV;
- Proportion protected at birth from neonatal tetanus; i.e. protection at birth (PAB);
- TT containing vaccine(s) administered to pregnant women during routine visits;
- Most recent TT supplementary immunization activities (SIA), age range and size of target population, vaccination coverage, vaccine presentation, and year of next planned activity;
- Number of adverse events following immunization (AEFI);
- Maternal and Neonatal Tetanus Elimination (MNTE) status (year of elimination);
- Influenza vaccine administered to pregnant women;
- Pertussis vaccine administered to pregnant women.

Immunization-associated activities

- Vitamin A supplementation.

For peer review only - http://bmjopen.bmj.com/site/about/guidelines.xhtml

Service delivery models

Routine maternal tetanus vaccination

- Policy content and coverage data;
- Existing delivery models, e.g. facility-based ANC and EPI/immunization services, outreach services, and regular and ad hoc health campaigns;
- Type(s) of vaccines administered, i.e. TT, Td, Tdap (adult formulation).

Integrated health campaigns for maternal tetanus vaccination

- Programme management and coverage data;
- Existing campaigns integrated with vaccination, e.g. deworming, vitamin A, malaria, nutrition;
- Past and future schedules of integrated health campaigns.

EPI, ANC or other organisation of maternal tetanus vaccination

- National level planning and management;
- Training (rationale, safety and AEFI surveillance) and supervision of vaccinators;
- Vaccine procurement and distribution;
- Monitoring and evaluation, i.e. records (ANC or EPI-based personal, clinic, or electronic), frequency of performance assessment, monitoring indicators, e.g. TT2+, PAB.

Funding for maternal tetanus vaccination programme

- Domestic and external funding.

Disease surveillance

- Maternal and neonatal tetanus, i.e. passive, sentinel, active, community-based;
- Other health indicators, i.e. congenital rubella syndrome, neonatal sepsis, neonatal mortality, maternal mortality, BCG at birth, OPV at birth, HBV at birth, other.

ANC capacity for maternal immunization

- Policy for ANC, i.e. number of visits, settings for ANC provision, i.e. government or private health facility/hospital, clinic, outreach programme;
- Any user fees for ANC and maternal immunization.

Vaccine safety surveillance

- Surveillance of AEFI following immunization in general and maternal immunization;
- Any available surveillance data.

Other maternal vaccines

- Routine maternal immunization, e.g. influenza, pertussis, or other;
- Programme management, i.e. EPI, ANC, or other responsible for planning, training, supervision, procurement, and distribution.

For peer review only - http://bmjopen.bmj.com/site/about/guidelines.xhtml

Country context

Overview

- Integration of ANC and EPI organisation, i.e. national level coordination of maternal immunization, representation of maternal and newborn health care experts in NITAG;
- National policy and action plan for maternal immunization, and respective targets, i.e. coverage, completeness and timeliness of reports, how and why targets are/are not met;
- Existence of national HMIS, completeness and mode of data collection, available data.

Funding of maternal tetanus immunization and ANC services

- Domestic and external funding of ANC services and maternal immunization, user fees for ANC and tetanus vaccination, and impact of funding situation on ANC and/or maternal immunization, e.g. procurement, logistics, training, mobilisation, and/or administration.

Human resources

- National and district level coordination and challenges for delivery.

Service delivery through ANC and the birth context

- Coverage and quality of ANC, i.e. staffing, coverage, precision of estimate of gestational age, counselling, prevention and interventions, referral systems, and outreach services;
- Challenges to ANC delivery, e.g. staffing, equipment, infrastructure;
- Information used for planning and prioritisation, e.g. coverage, staffing, funding, user needs;
- ANC records, i.e. verbal, written, electronic, and personal or facility-based, follow-up.

Tetanus vaccine delivery to pregnant women

Overview

- Type of vaccines delivered, i.e. TT, Td or other, frequency, any integration with ANC/EPI;
- Private providers of tetanus vaccination of pregnant women, available data;
- Existing quality of TT vaccine cold chain, and ANC services' capacity for vaccine storage;
- Current vaccination of pregnant women through ANC, staffing and challenges, e.g. infrastructure, cold chain, vaccine supply, skilled staff.

Vaccination of pregnant women outside ANC

- Primary, secondary and tertiary clinical settings for vaccination of pregnant women, staffing;
- Information used for planning and prioritisation of outreach services, e.g. ANC coverage, staffing, funding, user needs.

Recording of tetanus immunization during pregnancy

- Policy, guidelines, operating procedures, with attention to immunization history and dosage.

Maternal and neonatal tetanus surveillance

- Existing neonatal and maternal tetanus surveillance systems, available data;
- Frequency of reporting, integration with other surveillance systems;
- Existence, frequency and quality of monitoring.

For peer review only - http://bmjopen.bmj.com/site/about/guidelines.xhtml

Tetanus vaccine delivery to pregnant women, cont.

Surveillance of other diseases

- Maternal and neonatal mortality.

Vaccine safety surveillance systems

- Existence of training, surveillance of vaccination of pregnant women on AEFI, available data.

Other vaccines than tetanus in pregnancy

- Policy, partners, and delivery mechanisms for vaccines to pregnant women other than tetanus, e.g. influenza, pertussis, yellow fever, and meningococcus A, available data;
- Main barriers for introducing additional vaccines for pregnant women, by administration level;
- Potential interventions to support uptake of maternal vaccinations, e.g. elimination of user fees, client/provider communication, availability of medicines.

For peer review only - http://bmjopen.bmj.com/site/about/guidelines.xhtml

Supply side (health system)

Service delivery

- Integration of antenatal care (ANC) and Expanded Programme on Immunization;
- Accessibility, outreach services;
- Costs of services;
- Availability, supply chain;
- Quality and mode of delivery;
- Cultural appropriateness;
- Follow-up, e.g. mobile technology;
- Function of referral system.

Health care workers

- Education, professional skills;
- Workload, working conditions;
- Professional attitudes (non-discriminatory);
- Communication skills;
- Role of community health workers.

Information

- Actionable health information system;
- Demand side information campaigns.

Medical products, vaccines, technology

- Safety;
- Supply chain skills, documentation.

Financing

- Domestic, external funding;
- Devolution of health services planning and financing;
- Results-based approaches.

Leadership, governance

- Partnerships;
- Political priorities;
- Health system organisation, e.g. level of decentralisation;
- Accountability mechanisms;
- Community participation.

Demand side (pregnant women)

Socio-cultural and -economic factors

- Socio-economic status;
- General health literacy;
- Knowledge about maternal vaccination;
- Mobility, security;
- Personal characteristics, i.e. age, marital status, parity;
- Culture, religion.

Health systems interaction

- Reception of adequate information;
- Distance to health facility;
- Direct and indirect costs of services;
- Transport, infrastructure (safety, accessibility);
- Opportunity costs, i.e. time spent at facility;
- Clarity of procedures;
- Communication (trust);
- Non-discrimination;
- Community outreach.

BMJ Open

The Maternal Immunization and Antenatal Care Situation Analysis (MIACSA) study protocol: A multi-regional, cross-sectional analysis of maternal immunization delivery strategies to reduce maternal and neonatal morbidity and mortality

Journal:	*BMJ Open*
Manuscript ID	bmjopen-2018-024449.R1
Article Type:	Protocol
Date Submitted by the Author:	13-Oct-2018
Complete List of Authors:	Roos, Nathalie; Organisation mondiale de la Sante, Department of Maternal Newborn Child and Adolescent Health (MCA), Epidemiology Monitoring and Evaluation (EME)<br>Lambach, Philipp; Organisation mondiale de la Sante, Department of Immunization, Vaccines and Biologicals (IVB), Initiative for Vaccine Research (IVR)<br>Mantel, Carsten; MMGH Consulting GmbH; Robert Koch Institute, Department of Infectious Disease Epidemiology<br>Mason, Elizabeth; London School of Hygiene and Tropical Medicine<br>Munoz, Flor M.; Baylor Coll Med, Department of Pediatrics, Section of Infectious Diseases<br>Giles, Michelle; Monash University and Monash Health, Department of Obstetrics and Gynaecology<br>MIACSA, expert advisory panel group<br>Moran, A; World Health Organization, Department of Maternal Newborn Child and Adolescent Health (MCA), Epidemiology Monitoring and Evaluation (EME)<br>Hombach, Joachim; Organisation mondiale de la Sante, Department of Immunization, Vaccines and Biologicals (IVB), Initiative for Vaccine Research (IVR)<br>Diaz, Theresa; World Health Organization, Department of Maternal Newborn Child and Adolescent Health (MCA), Epidemiology Monitoring and Evaluation (EME)
**Primary Subject Heading**:	Global health
Secondary Subject Heading:	Global health, Public health
Keywords:	maternal immunization, maternal mortality, neonatal mortality, study protocol, maternal tetanus, neonatal tetanus

SCHOLARONE™
Manuscripts

The Maternal Immunization and Antenatal Care Situation Analysis (MIACSA) study protocol: A multi-regional, cross-sectional analysis of maternal immunization delivery strategies to reduce maternal and neonatal morbidity and mortality

Nathalie Roos[1]*, Philipp Lambach[2]*, Carsten Mantel[3], Elizabeth Mason[4], Flor M. Muñoz[5], Michelle L. Giles[6], MIACSA expert advisory panel group**, Allisyn C. Moran[1], Joachim Hombach[2]***, Theresa Diaz[1]***

1 Department of Maternal Newborn Child and Adolescent Health (MCA), Epidemiology Monitoring and Evaluation (EME), World Health Organization (WHO), Geneva, Switzerland

2 Department of Immunization, Vaccines and Biologicals (IVB), Initiative for Vaccine Research (IVR), WHO, Geneva, Switzerland

3 MMGH Consulting GmbH, Zürich, Switzerland and Department of Infectious Disease Epidemiology, Robert Koch Institute, Berlin, Germany

4 London School of Hygiene and Tropical Medicine, London, UK

5 Department of Pediatrics, Section of Infectious Diseases, Baylor College of Medicine, Houston, Texas, USA

6 Department of Obstetrics and Gynaecology, Monash University and Monash Health, Melbourne, VIC, Australia

* First authors

** The MIACSA expert advisory panel (EAP) consists of the following members: Flor M. Muñoz (chair), Michelle L. Giles (co-chair), Mercy Ahun, Martina Baye, Pradeep Haldar,

For peer review only - http://bmjopen.bmj.com/site/about/guidelines.xhtml

Matthews Mathai, and Stephen Hodgins. Observers to EAP meetings included: Carsten Mantel, Elizabeth Mason, Sonja Mertens, Jayani Pathirana, Sarah Rendell. Additional WHO experts included: Emily Wootton, Laura Nic Lochlainn, Ahmadu Yakubu, and Sara Rendell.

*** Last authors

Corresponding author: Dr Nathalie Roos, Department of Maternal Newborn Child and Adolescent Health (MCA), Epidemiology Monitoring and Evaluation (EME), WHO, Geneva, Switzerland, email: roosn@who.int, phone: +41 22 791 3191

Word count: 2,603

Key words: maternal immunization; maternal tetanus vaccination, maternal health; neonatal health; maternal tetanus; neonatal tetanus; maternal mortality; neonatal mortality; health care service delivery; antenatal care capacity; study protocol

For peer review only - http://bmjopen.bmj.com/site/about/guidelines.xhtml

ABSTRACT

Introduction. Maternal immunization (MI) is a safe and cost-effective way of preventing neonatal tetanus, and is one of several strategies that aim to reduce the high global rates of maternal and neonatal morbidity and mortality caused by vaccine-preventable diseases. Given the prospect of introducing new maternal vaccines in the near future, it is essential to identify and understand current policies, practices, and unmet needs for introducing and/or scaling up MI in low and middle income countries (LMICs).

Methods and analysis. The Maternal Immunization and Antenatal Care Situation Analysis (MIACSA) is a mixed methods, cross-sectional study that will collect data in four phases: (1) a review of global databases for selected health indicators in 136 LMICs; (2) a structured online survey directed at Maternal, Newborn, and Child Health (MNCH) and Expanded Programme on Immunization (EPI) focal points in all 136 LMICs; (3) semi-structured telephone interviews of 30 selected LMICs; and (4) 10 week-long country visits, including key informant interviews, health facility visits, and focus group discussions. The principal analyses will assess correlations between the various aspects of MI delivery strategies and proxy measures of health systems performance related to vaccine-preventable disease control. The primary outcome will be a typology of existing MI delivery models, and secondary outcomes will include country profiles of child and maternal health indicators, and an MI gaps and needs analysis.

Ethics and dissemination. The protocol was approved by the World Health Organization Ethics Review Committee. The results will be made available in a project report and submitted for publication in peer-reviewed journals that will be shared broadly among global health decision makers, researchers, product developers, and country-level stakeholders.

For peer review only - http://bmjopen.bmj.com/site/about/guidelines.xhtml

Registration. Not applicable.

For peer review only

For peer review only - http://bmjopen.bmj.com/site/about/guidelines.xhtml

ARTICLE SUMMARY

Strengths and limitations of this study

- The MIACSA study will provide a first time, comprehensive global overview and analysis of existing maternal immunization (MI) delivery strategies in low- and middle-income countries (LMICs).
- The results will provide evidence to inform the development of a typology of MI delivery approaches in LMICs, and identify capacity needs and key system changes, including policy adjustments required to introduce new maternal vaccines and/or scale up existing MI in LMICs.
- The study benefits from a mixed-methods design; a multidisciplinary approach leveraging policy-level, academic, and implementers' experience; multi-level data collection; a globally comprehensive analysis combined with in-depth information of a subsample of LMICs; inter-sectoral collaboration between MNCH and EPI programmes, and broad dissemination of results. Limitations include the small number of countries and health care facilities visited within each country included in the study, thus precluding generalization of country visit findings to a national level, and the fact that the end-users' perspective will be captured only indirectly through community health workers.

Data on maternal immunization service delivery collected through an online survey targeting all low and middle income countries, will be analysed within the limitations of validity of data collected.

For peer review only - http://bmjopen.bmj.com/site/about/guidelines.xhtml

INTRODUCTION

Vaccine-preventable diseases are a major cause of global child morbidity and mortality, particularly in low- and middle-income countries (LMICs).[1] Since the 1990s, public health interventions have more than halved under-five childhood mortality; however, reduction of stillbirths and of neonatal mortality (death in the first 28 days of life) has been slower.[2] This is in part due to the fact that most vaccines cannot be administered to newborns, who, being unable to develop protective responses due to limitations in their immune system, are left particularly vulnerable to infectious diseases. Vaccination of pregnant women, or maternal immunization (MI), has proven to be an effective strategy to reduce neonatal tetanus, and is a potential strategy to reduce the burden of other vaccine-preventable diseases in mothers and infants. Thus, MI is one of several strategies that aim to reach the third sustainable development goal of ending preventable maternal and newborn deaths.[3-5]

Studies have shown that MI can effectively protect the mother, as well as her child, through transplacental transfer of maternal immunoglobulin G (IgG) to the foetus.[6, 7] The Maternal and Neonatal Tetanus Elimination (MNTE) initiative has led the way in the implementation of MI, combining at least two doses of tetanus toxoid containing vaccine (TTCV) during pregnancy (TT2+) with the promotion of hygienic delivery and clean cord care practices, as well as vaccination of children and women of reproductive age, to eliminate maternal and neonatal tetanus as a public health problem. Between the late 1980s and 2015, the MNTE initiative reduced global tetanus-related neonatal mortality by 96%.[8]

For peer review only - http://bmjopen.bmj.com/site/about/guidelines.xhtml

TTCV and inactivated influenza vaccines are considered safe and effective for use during pregnancy,[9] and are recommended for pregnant women by the World Health Organization (WHO).[6, 10-13] New vaccines, several of which are under development and evaluation, target other important pathogens, such as group B streptococcus (GBS), and respiratory syncytial virus (RSV), and may provide safe and cost-effective protection of mothers and their infants through MI in the future.[14-18]

In order to identify the challenges of implementing current and new vaccines for MI, a better understanding is needed of the capabilities and limitations of existing delivery platforms, such as antenatal care (ANC) services and the Expanded Programme on Immunization (EPI).[19] The capacity of ANC services to deliver vaccines to pregnant women will require thorough assessment, as globally only 62% of women benefit from at least four ANC visits, i.e. the proportion of pregnant women who received 4 or more ANC visits during their last pregnancy (ANC4+), and in Sub-Saharan Africa and South Asia, ANC4+ coverage is only 52% and 46%, respectively.[20] Delivering vaccinations and other essential interventions to women at the necessary timely intervals during pregnancy, as well as documenting the coverage and outcomes of such interventions, requires a robust ANC platform with sufficient personnel and resources.[21]

WHO recommends that pregnant women living in endemic areas are sufficiently immunised against tetanus in order to protect the women and their newborn infants. MI with TTCV is routine in many countries;[22, 23] however, progress of tetanus vaccination in LMICs has faced challenges leading to delays in elimination, and uptake among pregnant women of other

For peer review only - http://bmjopen.bmj.com/site/about/guidelines.xhtml

vaccines, such as influenza and pertussis vaccines, has been low. As a part of EPI services, routine tetanus immunization during pregnancy has been complemented with supplementary vaccination activities in a majority of countries in order to reach high coverage and achieve MNTE goals. A better understanding of MI in the context of both ANC and EPI, including implementation of guidelines and policies, ministerial responsibilities at national and subnational levels, vaccine management including cold chain and logistics, vaccine administration, staff capacity, social mobilisation, vaccine acceptance, and assessment of vaccine safety, may help identify service delivery challenges as well as opportunities to optimise current and future MI efforts.[24]

Closer collaboration between ANC and EPI services could provide a unique and cost-effective opportunity to further strengthen preventive health care measures for women and children under each programme, by reducing missed opportunities for immunization, including MI, as well as reinforcing the delivery of essential health care services.

In view of recent product and policy developments, the WHO, supported by the Bill and Melinda Gates Foundation, aims to identify the knowledge gaps in MI delivery strategies by mapping the strengths and challenges of existing ANC and immunization services for pregnant women in LMICs through the Maternal Immunization and Antenatal Care Situation Analysis (MIACSA) project. The results will provide the evidence for a typology of MI delivery models, as well as identify the capacity needs and key system changes required to introduce new maternal vaccines and/or strengthen vaccine delivery for MI in LMICs. Ultimately, the project aims to identify and

For peer review only - http://bmjopen.bmj.com/site/about/guidelines.xhtml

111 understand current MI and ANC related policies, practices and the need for strengthening both immunization and maternal child health care services , and how they could accommodate new MI vaccines.

For peer review only

For peer review only - http://bmjopen.bmj.com/site/about/guidelines.xhtml

METHODS AND ANALYSIS

Patient and Public Involvement.

The development of the research questions were influenced by an interdisciplinary group of international experts for the MIACSA project. The project did not include patients, but restricted itself to national level program managers and health facilities where health workers responded to interviews in their professional capacity.

Study design and data collection

Between November 2016 and December 2018, a mixed-methods, cross-sectional study will be carried out in four phases to assess key health system features related to the implementation of MI (Figure 1). An expert advisory panel (EAP) consisting of specialists in immunization, maternal and neonatal health, MI implementation and social sciences, will provide technical advice on the study design, the development of research questions and surveys, the data collection methods, and the results interpretation. In addition to following WHO standards for global monitoring surveys, all data collection tools and standard operating procedures will be reviewed and endorsed by the EAP. The surveys and country visits will be conducted in local languages when needed.

Desk review of global data (Data collection phase 1). The first phase will consist of collecting key health indicators of LMICs to create outlines of country profiles, focusing on ANC and EPI services. A desk review of pre-defined health indicators (Figure 2) from 136 LMICs will be

For peer review only

For peer review only - http://bmjopen.bmj.com/site/about/guidelines.xhtml

137 conducted from existing global data sources, including Demographic and Health Surveys
138 (DHS)/Multiple Indicator Cluster Surveys (MICS), WHO/United Nations Children's Fund
139 (UNICEF) estimates of national immunization coverage, WHO/UNICEF Joint Reporting Forms
140 (JRF), MNTE reports, and WHO Maternal, Newborn, Child, and Adolescent Health (MNCAH)
141 policy survey. The indicators will focus on governance and policy environment, health systems
142 performance, and immunization activities, including MI. Data from phase 1 will be compiled in a
143 database for analysis of the study's research questions, and will inform the selection of countries
144 for phase 3 (see below).

145

146 **Global online survey (Data collection phase 2).** The country profiles established in phase 1
147 will be completed and, if needed, updated by an online survey with WHO Regional Offices,
148 national Ministry of Health (MoH) focal points from Maternal, Newborn and Child Health
149 (MNCH) and EPI programmes and their WHO Country Office counterparts in all LMICs, using
150 a structured questionnaire (Figure 3). Data will be collected on service delivery models of
151 maternal tetanus vaccination, including delivery platforms, programme funding, disease
152 surveillance, and vaccine safety surveillance. Data on maternal vaccines other than tetanus will
153 be included when pertinent. A draft questionnaire will be piloted in advance of the survey. Non-
154 responders will be followed-up by telephone and email. Revisions following queries on missing,
155 erroneous, or inconsistent data will be done at country level.

156

157 **Telephone interviews (Data collection phase 3) .** In order to understand how existing health
158 care delivery services could be adapted to implement MI beyond tetanus immunization, further

For peer review only - http://bmjopen.bmj.com/site/about/guidelines.xhtml

data will be collected on delivery platforms for maternal tetanus vaccination in LMICs. In-depth telephone interviews will be conducted with EPI and MNCH programme officers responsible for MI at the MoH in a sample of 30 countries, using a semi-structured questionnaire (Figure 4). The countries will be selected based on performance of MI as assessed by coverage of maternal TTCV and ANC, geographic representation, and recommendations from WHO Regional Offices on MI priorities. The countries will be stratified into four groups; high and low maternal tetanus vaccination performance measured as protection at birth (PAB), i.e. the proportion of newborns protected at birth against neonatal tetanus, with a cut-off of 90%, and high and low ANC performance (with a cut-off of the median ANC4+ coverage in countries with available data). PAB was identified as a more reliable proxy measure than TT2+, as the issue of not including already vaccinated women in the numerator used for estimating the latter indicator would be avoided. The PAB cut-off level was set based on the target required to attain and sustain MNTE, whereby >80% of pregnant women are immunized against tetanus. The country selection will include a representation of all MI delivery models and WHO regions, with a focus on Africa and South-East Asia where maternal and neonatal mortality are highest, and will ensure inclusion of high performing countries in order to include likely early adopters of new maternal vaccines and learning cases of best practices.

The interviews will collect data on the policy, governance, and funding environment for EPI and ANC programmes, ANC delivery, and maternal tetanus vaccination including monitoring and evaluation of results. The questionnaire will be shared with WHO country office focal points and MoH MNCH and EPI managers for compilation in advance of the teleconference, allowing for discussion and clarification when needed during the actual interview. Responses will be recorded

For peer review only - http://bmjopen.bmj.com/site/about/guidelines.xhtml

using standard data entry procedures, and may be voice-recorded if consent is obtained by the interviewees. Any discordant responses will be attempted to be resolved by consensus, and incomplete responses will be followed up. A summary of key findings will be shared with the participants to confirm the responses were correctly captured.

Country visits (data collection phase 4). Finally, in-country visits will be conducted in order to collect data on MI from key decision-makers and implementers at every level of the health care system, as well as to determine actual delivery, capacity and coordination of ANC and EPI services, on both supply and demand sides of the health care services (Figure 5). Ten countries will be selected based on high, medium, or low performance of MI systems as assessed by PAB and TT2+, a range of different MI delivery models (e.g. degree of coordination between EPI and ANC in MI delivery), and agreement by senior national and subnational MNCH and EPI staff for study visits. The final country selection will ensure representation of the range of MI delivery models, and will include high-performing countries, MNTE priority countries, and countries with high ANC4+ coverage. Site visits will include ANC and EPI sites and session observations, focus group discussions, and in-depth interviews. The week-long visits will be piloted in two countries to adjust and refine the data collection tools and the standard operating procedures, and data from these two countries will be included in the final analysis.

An initial joint focus group discussion will be held with national-level stakeholders, followed by key informant interviews with stakeholders pertinent to MI, ANC, and EPI services at subnational levels of the health care system, including decision and policy makers, technical and

For peer review only

financial parties, and civil society, such as non-governmental organisations. The study will aim to conduct a total of twelve health facility visits taking into account a balance of geographical locations, urban and rural areas, and - if possible - different types of health facilities (e.g. small and larger health units). The country visits will be concluded with an on-site debriefing and joint data analysis with MoH MNCH and EPI focal points and other main country-level stakeholders. End-users, i.e. pregnant women, will not be interviewed as it would require a separate study design; however, their perspective will be indirectly included through the participation of community health workers at stakeholder meetings.

Data analysis plan

The cross-sectional data analyses will be carried out over four data collection 4 phases (desk review of global data, online questionnaire and indepth country interviews, and country visits). The first three will yield quantitative data. The last two data collection phases will also provide an in-depth qualitative analyses of data collected from a select number of countries. Below we describe the analyses for each phase.

Desk review of global data(phase 1)

The MIACSA project will conduct a desk review of global databases (Joint reporting form (JRF), United Nations (UN) mortality reports, Demographic health survey (DHS), Multiple Cluster Indicators Survey (MICS), WHO MNCAH policy survey database, maternal and neonatal tetanus elimination (MNTE) database, WHO/UNICEF Estimates of National Immunization Coverage (WUENIC)) targeting 136 low- and middle income countries (LMIC). The primary outcome variable (dependant variable) to asses MI performance will be PAB (cut-

For peer review only - http://bmjopen.bmj.com/site/about/guidelines.xhtml

off level <90% and ≥90%) and the independent variables will include country economic level, immunization coverage, mortality, service coverage, available ANC and vaccination policies and availability of a national immunization advisory committee (Figure 2).

We will first asses the database for completeness of data. We will also conduct a sensitivity analysis based on imputation of data based on available predictors for countries with missing data on PAB. Results from the complete case analysis will be compared with the sensitivity analysis to explore bias due to missing data.

We will conduct bivariate analyses to assess whether the dependant variables are associated with the independent variables. We will also do multivariable analyses within subgroups, since vaccinations may differ by other factors (e.g., WHO Regions; GAVI status; World Bank income level ; MNTE; female literacy rate).

For continuous variables we will first assess the normality using the Shapiro-Wilk test. If needed, we will make appropriate transformations to normalize the data or group them into categories as necessary. We will then compare the distributions of the variables by groups with two-sided chi-square (categorical variables) or t-tests (continuous variables) or the equivalent non-parametric tests (e.g., Fisher's exact or Wilcoxon/Kruskal-Wallis), as appropriate. A two-sided p-value of 0.05 will be considered as significant.

To create a multivariable model, we will include all variables that are significantly associated with the dependant variable and those variables which have shown association within the available literature. We will then asses for collinearity and remove one of the variables if collinearity is found. . We will also assess for interactions and will create interaction terms to be included in the model if any interactions are found. Both forward and backward elimination will

For peer review only - http://bmjopen.bmj.com/site/about/guidelines.xhtml

be conducted to assess the goodness of fit and create the final model.

Global online survey (phase 2)

The variables are based on the online survey as described above. For a summary of the included components see Figure 3. Data from the online survey will be checked for completeness and consistency and coded to reflect skip patterns. The complete data set for analyses will include PAB from phase 1 (desk review database) and will be linked with the database containing responses for the global online questionnaire.. Descriptive analyses will be conducted including summary measures. Bivariate analyses will be conducted to assess the associations between the questionnaire variables and the dependant variable PAB and the significance of the relationship will be tested with Fisher's Exact test. Logistic regression models will be used to assess the relationship between the responses in the online questionnaire and high coverage of PAB >=90% independently . These models will be created as described in phase 1. A two-sided p-value of 0.05 will be considered as significant.

Telephone conferences (quantitative analyses, phase 3)

The primary objective is to provide descriptive information about maternal immunization services and its organization (Figure 4).

Data from this phase will be checked for completeness and consistency and coded to reflect skip patterns. Descriptive analyses will be conducted including summary measures.

Qualitative analyses based on country visits and telephone conferences (phase 4)

For peer review only - http://bmjopen.bmj.com/site/about/guidelines.xhtml

Ten countries will be visited to conduct qualitative interviews at national and subnational level. Resulting qualitative information and from these visits as well as from telephone interview conducted in the previous phase will be used in a thematic analysis applied by trained qualitative data analysts to the following qualitative data sources: comments and free-text responses to telephone interview questions (phase 3), semi-structured interviews with community health workers (phase 4), comments and free-text response to stakeholder and facility manager interviews (phase 4), comments provided during debrief sessions with national level stakeholders in-country (phase 4). Thematic analysis will be applied to intra-case and cross-case analysis. First, an intra-case analysis will organize and reduce qualitative findings within each country along two criteria: (1) relevance of finding to research questions and (2) relative frequency of finding across data sources. Second, a cross-case analysis will organize findings across countries into themes generated from research questions and sub-themes generated from grounded analysis of data collected. Two qualitative data analysts will co-organize and reduce intra-country findings. For cross-country findings, qualitative analysts will independently generate themes and sub-themes for cross-case analysis and will then resolve any inter-coder divergence in themes and sub-themes based on relevance of theme to data source, relevance of theme to research questions and robustness of theme relative to alternative themes. See figure 4 and 5 for the included components.

Consolidated data analysis

To inform the development of a typology of MI delivery models approaches in LMICs quantitative and qualitative data analysis results will be consolidated in a global analysis of MI and ANC service delivery approaches in countries as well as individual country profiles that

For peer review only - http://bmjopen.bmj.com/site/about/guidelines.xhtml

295 shall support countries to conduct self-assessments of their MI and ANC systems strengths and capacity gaps. Based on the advice of the project's advisory group, a checklist approach will be considered to provide a profiling for countries with sufficient data available, including indicators on policy and governance, financing, programme management, service delivery systems and demand side issues. Ultimately, such a profiling shall help countries and other MI stakeholders to identify the needs for targeted support to strengthen existing MI programmes or to reach readiness to introduce future programmes.

Limitations

The data analyses will take into account the limitations of the study, including the reliability of the selected outcome measures, i.e. PAB, potential biases introduced by the limited number of countries for which in-depth information will be available, i.e. through telephone interviews and in-country visits, selective sampling of in-country site visit locations, missing data, and the fact that the end-users' perspective will be captured only indirectly through facility and community based health workers.

For peer review only - http://bmjopen.bmj.com/site/about/guidelines.xhtml

ETHICS AND DISSEMINATION

Ethical considerations. The first three phases of the study are exempt from ethical permission as participants will provide information on operations and administration of public health services on a purely professional basis, and without disclosure of person-identifiable information. The protocol for the country visits was approved by the WHO Research Ethics Review Committee (ERC.0002908).

Country ownership will be ensured through transfer of responsibility for the data provision to in-country focal points, and by joint, on-site analysis of the data collected during the country visits with the main stakeholders. The study aims to contribute to the evidence needed to ensure more equitable access to high-impact global health interventions, such as MI.[25]

Data management and dissemination

The data will be managed and analysed by data clerks who were not part of the data collection. Anonymised data from surveys and key informant interviews, excluding any confidential information as identified by the in-country focal points, will be uploaded to a publicly available data repository hosted by the WHO. Recordings from country interviews will be transcribed before the qualitative analyses and destroyed at completion of the data analyses.

The results will be submitted for publication in peer-reviewed journals, as well as in a MIACSA project report that will be shared widely with global health decision makers, researchers, product

For peer review only - http://bmjopen.bmj.com/site/about/guidelines.xhtml

developers, and implementers. The report and/or specific aspects of the project, will be presented at international stakeholder meetings, with the ultimate aim to establish a knowledge network of countries exploring MI implementation strategies. Further, the results will be shared through summaries on the WHO website and in public fora.

To ensure wide distribution of the project findings to the international scientific community and national stakeholders involved in maternal immunization, findings will be also shared at the end of the project through a a large stakeholder convening. At this meeting, key aspects of maternal tetanus vaccination service delivery mechanisms and antenatal care capacities identified in select countries will be discussed to enable exchange of lessons learnt between select participating countries and to discuss generalizable lessons learnt that may improve maternal immunization service delivery through an integrated platform considering Immunization and Maternal Child Health Care mechanisms.

Dissemination of the MIACSA results will aim to provide advice on best practices, policy requirements, capacity needs, and health system changes needed for successful introduction and integration of new maternal vaccines into national health systems, including ANC and EPI services, in LMICs.

For peer review only

For peer review only - http://bmjopen.bmj.com/site/about/guidelines.xhtml

REFERENCES

1. UNICEF WHO, and World Bank Group. Levels and Trends in Child Mortality: Report 2017. New York, 2017.

2. Lawn JE, Blencowe H, Oza S, et al. Every Newborn: progress, priorities, and potential beyond survival. *Lancet* 2014;384:189-205.

3. Sobanjo-Ter Meulen A, Abramson J, Mason E, et al. Path to impact: A report from the Bill and Melinda Gates Foundation convening on maternal immunization in resource-limited settings; Berlin - January 29-30, 2015. *Vaccine* 2015;33:6388-95.

4. You D, Hug L, Ejdemyr S, et al. Global, regional, and national levels and trends in under-5 mortality between 1990 and 2015, with scenario-based projections to 2030: a systematic analysis by the UN Inter-agency Group for Child Mortality Estimation. *Lancet* 2015;386:2275-86.

5. WHO. Trends in maternal mortality: 1990 to 2015. Estimates by WHO, UNICEF, UNFPA, World Bank Group and the United Nations Population Division. Geneva: World Health Organization, 2015.

6. Englund JA, Mbawuike IN, Hammill H, et al. Maternal immunization with influenza or tetanus toxoid vaccine for passive antibody protection in young infants. *J Infect Dis* 1993;168:647-56.

7. Puck JM, Glezen WP, Frank AL, et al. Protection of infants from infection with influenza A virus by transplacentally acquired antibody. *J Infect Dis* 1980;142:844-9.

8. WHO. http://www.who.int/immunization/diseases/MNTE_initiative/en/ Accessed 22 January 2018.

For peer review only

For peer review only - http://bmjopen.bmj.com/site/about/guidelines.xhtml

9. Keller-Stanislawski B, Englund JA, Kang G, et al. Safety of immunization during pregnancy: a review of the evidence of selected inactivated and live attenuated vaccines. *Vaccine* 2014;32:7057-64.

10. WHO. Safety of immunization during pregnancy: A review of the evidence. World Health Organization. Geneva, 2014.

11. Hviid A, Svanstrom H, Molgaard-Nielsen D, et al. Association Between Pandemic Influenza A(H1N1) Vaccination in Pregnancy and Early Childhood Morbidity in Offspring. *JAMA Pediatr* 2017;171:239-48.

12. Vaccines against influenza WHO position paper - November 2012. *Wkly Epidemiol Rec* 2012;87:461-76.

13. Eick AA, Uyeki TM, Klimov A, et al. Maternal influenza vaccination and effect on influenza virus infection in young infants. *Arch Pediatr Adolesc Med* 2011;165:104-11.

14. Russell LB, Kim SY, Cosgriff B, et al. Cost-effectiveness of maternal GBS immunization in low-income sub-Saharan Africa. *Vaccine* 2017;35:6905-14.

15. Donders GG, Halperin SA, Devlieger R, et al. Maternal Immunization With an Investigational Trivalent Group B Streptococcal Vaccine: A Randomized Controlled Trial. *Obstet Gynecol* 2016;127:213-21.

16. Chaithongwongwatthana S, Yamasmit W, Limpongsanurak S, et al. Pneumococcal vaccination during pregnancy for preventing infant infection. *Cochrane Database Syst Rev* 2015;1:CD004903.

17. PATH. Advancing maternal immunization (AMI) http://sites.path.org/cvia/our-disease-targets/respiratory-syncytial-virus/advancing-maternal-immunization-ami/2017 Accessed 22 January 2018.

For peer review only - http://bmjopen.bmj.com/site/about/guidelines.xhtml

393 18. Glenn GM, Smith G, Fries L, et al. Safety and immunogenicity of a Sf9 insect cell-derived
394 respiratory syncytial virus fusion protein nanoparticle vaccine. *Vaccine* 2013;31:524-32.
395 19. Singh K, Story WT, Moran AC. Assessing the Continuum of Care Pathway for Maternal
396 Health in South Asia and Sub-Saharan Africa. *Matern Child Health J* 2016;20:281-9.
397 20. UNICEF. Antenatal care coverage: At least four visits
398 https://data.unicef.org/topic/maternalhealth/antenatal-care/#2017 Accessed 10 April
399 2017.
400 21. Hodgins S, D'Agostino A. The quality-coverage gap in antenatal care: toward better
401 measurement of effective coverage. *Glob Health Sci Pract* 2014;2:173-81.
402 22. Pecenka C, Munthali S, Chunga P, et al. Maternal influenza immunization in Malawi:
403 Piloting a maternal influenza immunization program costing tool by examining a
404 prospective program. *PLoS One* 2017;12:e0190006.
405 23. McMorrow ML, Emukule GO, Obor D, et al. Maternal influenza vaccine strategies in Kenya:
406 Which approach would have the greatest impact on disease burden in pregnant women
407 and young infants? *PLoS One* 2017;12:e0189623.
408 24. Nesin M, Read J, Koso-Thomas M, et al. Maternal Immunization: Current status and future
409 prospects. *Vaccine* 2015;33:6371.
410 25. Verweij M, Lambach P, Ortiz JR, et al. Maternal immunisation: ethical issues. *Lancet Infect*
411 *Dis* 2016;16:e310-e14.

412

For peer review only

For peer review only - http://bmjopen.bmj.com/site/about/guidelines.xhtml

AUTHORS' CONTRIBUTIONS

NR and PL designed the study with inputs from TD and JH; CM, EM, FMM, MLG, TD, JH, AM and the MIACSA expert advisory panel group drafted the protocol with NR and PL; and all authors reviewed and approved the final manuscript version.

ACKNOWLEDGMENTS

The investigators wish to thank the EPI and MNCH focal points at the regional and national WHO offices for their helpful contributions to the planning of the study, and the external specialists of the Expert Advisory Panel for their valuable advice and guidance on development of the protocol methodology. The authors also wish to thank Dr. Peter Mark Jourdan for drafting and editing the manuscript. There was no involvement from the public or patients in the project development.

FUNDING

This work was supported by the Bill and Melinda Gates Foundation, grant number OPP1156378.

COMPETING INTERESTS

The authors declare no competing interests.

For peer review only

For peer review only - http://bmjopen.bmj.com/site/about/guidelines.xhtml

FIGURE LEGENDS

Figure 1. Key health system features studied by the MIACSA project.

EPI = Expanded Programme on Immunization, ANC = antenatal care, AEFI = adverse events following immunization, TT = tetanus toxoid.

Figure 2. Study phase 1: List of indicators for the review of global databases.

CES = coverage evaluation survey, WUENIC = World Health Organization (WHO)/United Nations Children's Fund (UNICEF) estimates of national immunization coverage, BCG = Bacillus Calmette-Guérin vaccine, DPT1 = first dose of diphtheria-pertussis-tetanus vaccine, DPT3 = third dose of diphtheria-pertussis-tetanus vaccine, HepB1 = first dose of hepatitis B vaccine, HepB3 = third dose of hepatitis B vaccine, Hib1 = first dose of *Haemophilus influenzae* type B vaccine, Hib3 = third dose of *H. influenzae* type B vaccine, MCV1 = first dose of measles-containing vaccine, MCV2 = second dose of measles-containing vaccine, PcV1 = first dose of pneumococcal conjugate vaccine, PcV3 = third dose of pneumococcal conjugate vaccine, Pol1 = first dose of polio-containing vaccine, Pol3 = third dose of polio-containing vaccine, TT1 = first dose of tetanus toxoid vaccine, TT1+ = at least one dose of tetanus toxoid vaccine, TT2 = second dose of tetanus toxoid vaccine, TT2+ = at least two doses of tetanus toxoid vaccine, TT3 = third dose of tetanus toxoid vaccine, TT4 = fourth dose of tetanus toxoid vaccine, TT5 = fifth dose of tetanus toxoid vaccine, RCV1 = first dose of rubella-containing vaccine, RotaC = second or third dose of rotavirus vaccine depending on number of doses recommended in national schedule, YFV = yellow fever vaccine.

For peer review only

For peer review only - http://bmjopen.bmj.com/site/about/guidelines.xhtml

For peer review only

For peer review only - http://bmjopen.bmj.com/site/about/guidelines.xhtml

Figure 3. Study phase 2: Variables collected from online survey of 136 LMICs.

A structured questionnaire will be used to determine which service delivery platforms are in place for tetanus vaccination of pregnant women in low- and middle-income countries (LMICs), and to understand how existing health services could be adapted to implement maternal immunization beyond tetanus vaccination. Internal validation questions are incorporated in the questionnaire, and sources of data are requested, i.e. if administrative data or personal estimates. ANC = antenatal care, EPI = Expanded Programme on Immunization, TT = tetanus toxoid, Td = tetanus-diphtheria, Tdap = tetanus-diphtheria-acellular pertussis, AEFI = adverse events following immunization, TT2+ = at least 2 doses of tetanus toxoid vaccine during pregnancy, PAB = protection at birth, BCG = Bacillus Calmette-Guérin vaccine, OPV = oral polio vaccine, HBV = hepatitis B vaccine.

Figure 4. Study phase 3: Variables collected from interviews of 30 selected LMICs.

A semi-structured questionnaire will be used to assess the preparedness of antenatal care services for introducing (additional) immunizations for pregnant women in selected low- and lower-middle income countries, and to understand the strengths and weaknesses of current immunization to guide future planning. Internal validation questions are incorporated and probing for further details will be done when deemed necessary by the interviewer(s). Sources of data provided are requested, i.e. if administrative data or personal estimates. ANC = antenatal care, EPI = Expanded Programme on Immunization, NITAG = National Immunisation Technical Advisory Group, HMIS = Health management Information System, TT = tetanus toxoid, Td = tetanus-diphtheria, AEFI = adverse events following immunization.

For peer review only - http://bmjopen.bmj.com/site/about/guidelines.xhtml

Figure 5. Study phase 4: Country study analysis framework for 10 country visits.

Key informant interviews, health facility visits, and focus group discussions will enable observation and collection of further data on the variables from the previous study phases, in particular at different levels of the health care system, and of socio-cultural and socio-economic factors. End-users, i.e. pregnant women, will not be interviewed as it would require a separate study design, and their perspective will be indirectly included through the participation of community health workers at stakeholder meetings.

For peer review only

For peer review only - http://bmjopen.bmj.com/site/about/guidelines.xhtml

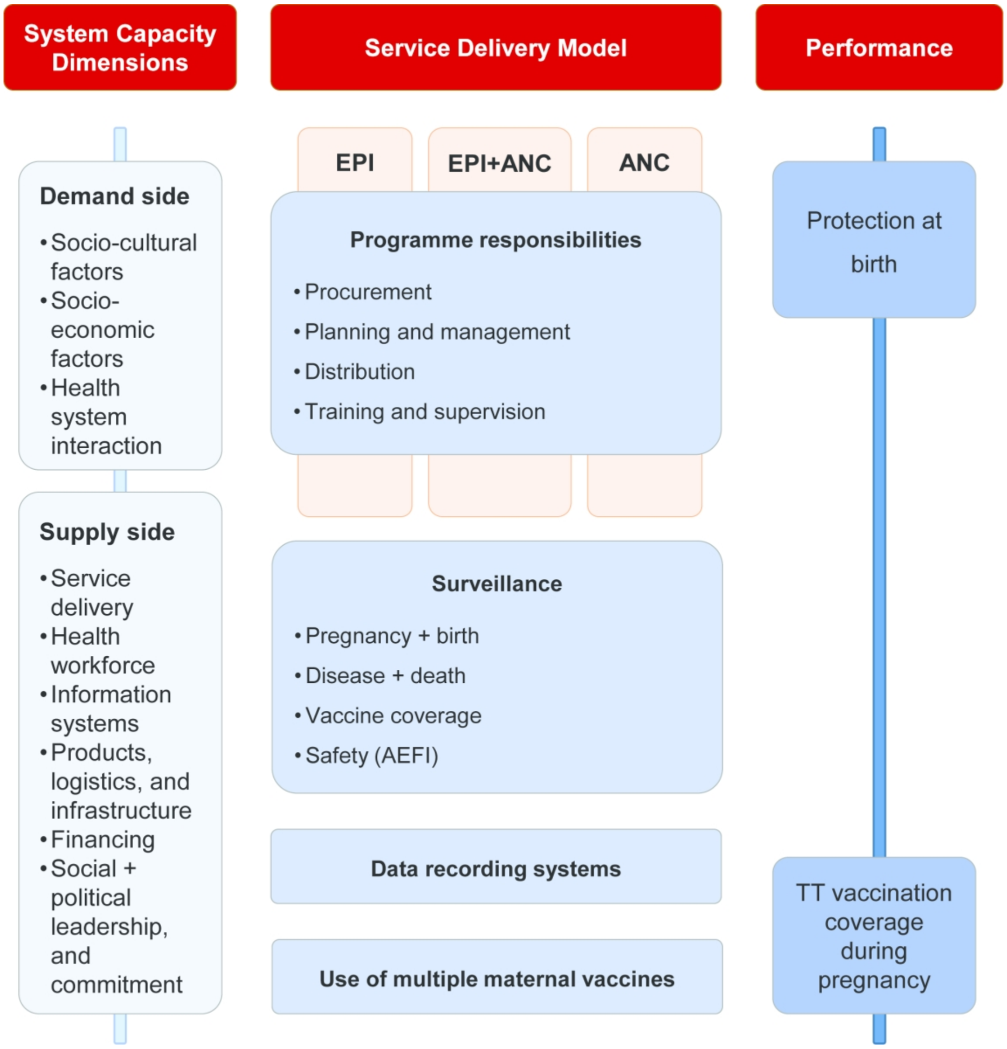

EPI = Expanded Programme on Immunization, ANC = antenatal care, AEFI = adverse events following immunization, TT = tetanus toxoid.

General

- World Bank income classification;
- Female literacy rate.

Health systems

General

- Health systems classification;
- Birth cohort (most recent year of available data);
- Target population of pregnant women.

Governance and policy environment

- Percentage of total expenditure on routine immunization financed by government funds;
- Existence of national immunization technical advisory group (NITAG);
- National policy on minimum antenatal care (ANC) visits;
- Eligibility for global vaccine alliance (GAVI) support.

Health systems performance

- Maternal, neonatal, and infant mortality;
- Stillbirth rates;
- Physician and midwife densities;
- Institutional deliveries;
- Coverage of a minimum of four ANC visits (ANC4+).

Vaccination, including maternal immunization

- Number of confirmed tetanus and neonatal cases;
- Coverage of at least 2 doses of TT vaccine during pregnancy (TT2+);
- Tetanus toxoid (TT) vaccine as a proportion of CES and WUENIC vaccines, i.e. BCG, DPT1, DPT3, HepB1, HepB3, Hib1, Hib3, MCV1, MCV2, PAB, PcV1, PcV3, Pol1, Pol3, TT1, TT1+, TT2, TT2+, TT3 TT4, TT5, RCV1, RotaC, YFV;
- Proportion protected at birth from neonatal tetanus; i.e. protection at birth (PAB);
- TT containing vaccine(s) administered to pregnant women during routine visits;
- Most recent TT supplementary immunization activities (SIA), age range and size of target population, vaccination coverage, vaccine presentation, and year of next planned activity;
- Number of adverse events following immunization (AEFI);
- Maternal and Neonatal Tetanus Elimination (MNTE) status (year of elimination);
- Influenza vaccine administered to pregnant women;
- Pertussis vaccine administered to pregnant women.

Immunization-associated activities

- Vitamin A supplementation.

Figure 2. Study phase 1: List of indicators for the review of global databases. CES = coverage evaluation survey, WUENIC = World Health Organization (WHO)/United Nations Children's Fund (UNICEF) estimates of national immunization coverage, BCG = Bacillus Calmette-Guérin vaccine, DPT1 = first dose of diphtheria-pertussis-tetanus vaccine, DPT3 = third dose of diphtheria-pertussis-tetanus vaccine, HepB1 = first dose of hepatitis B vaccine, HepB3 = third dose of hepatitis B vaccine, Hib1 = first dose of Haemophilus influenzae type B vaccine, Hib3 = third dose of H. influenzae type B vaccine, MCV1 = first dose of measles-containing vaccine, MCV2 = second dose of measles-containing vaccine, PcV1 = first dose of pneumococcal conjugate vaccine, PcV3 = third dose of pneumococcal conjugate vaccine, Pol1 = first dose of polio-containing vaccine, Pol3 = third dose of polio-containing vaccine, TT1 = first dose of tetanus toxoid vaccine, TT1+ = at least one dose of tetanus toxoid vaccine, TT2 = second dose of tetanus toxoid vaccine, TT2+ = at least two doses of tetanus toxoid vaccine, TT3 = third dose of tetanus toxoid vaccine, TT4 = fourth dose of tetanus toxoid vaccine, TT5 = fifth dose of tetanus toxoid vaccine, RCV1 = first dose of rubella-containing vaccine, RotaC = second or third dose of rotavirus vaccine depending on number of doses recommended in national schedule, YFV = yellow fever vaccine.

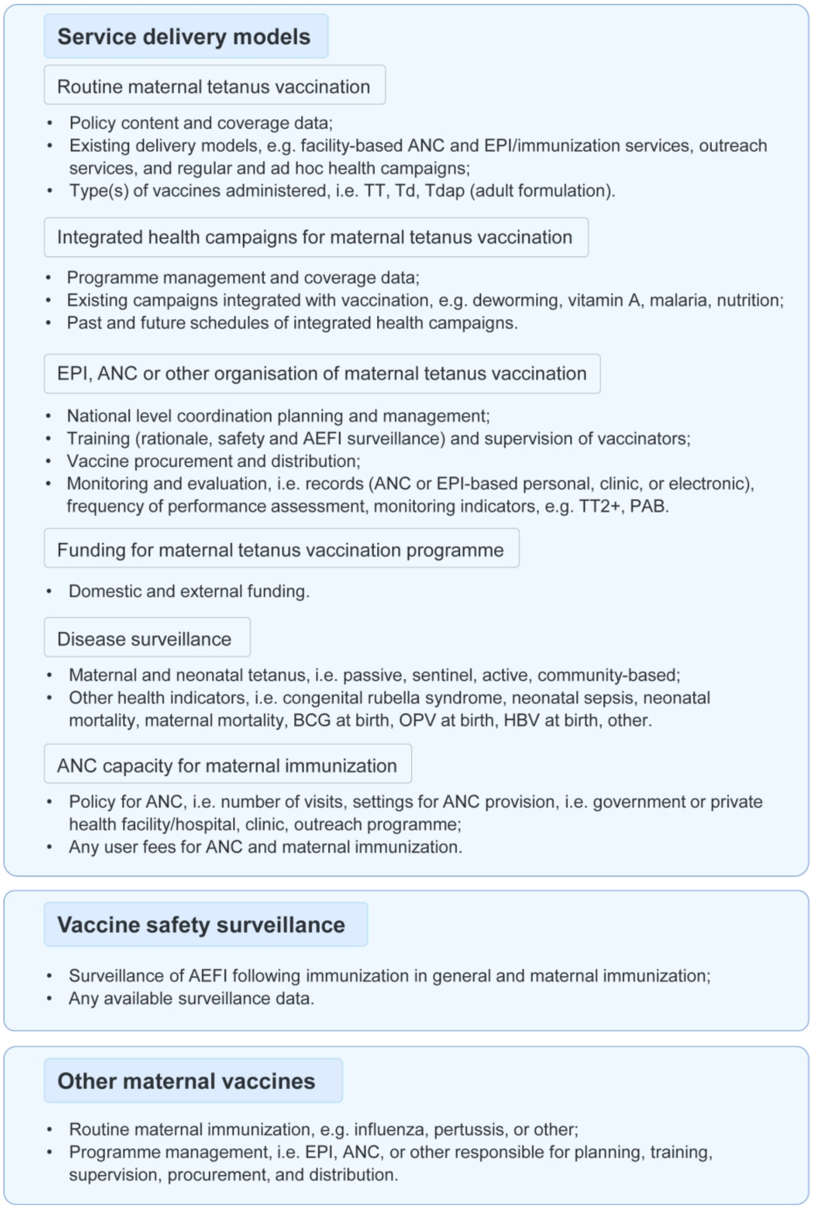

A structured questionnaire will be used to determine which service delivery platforms are in place for tetanus vaccination of pregnant women in low- and middle-income countries (LMICs), and to understand how existing health services could be adapted to implement maternal immunization beyond tetanus vaccination. Internal validation questions are incorporated in the questionnaire, and sources of data are requested, i.e. if administrative data or personal estimates. ANC = antenatal care, EPI = Expanded Programme on Immunization, TT = tetanus toxoid, Td = tetanus-diphtheria, Tdap = tetanus-diphtheria-acellular pertussis, AEFI = adverse events following immunization, TT2+ = at least 2 doses of tetanus toxoid vaccine during pregnancy, PAB = protection at birth, BCG = Bacillus Calmette-Guérin vaccine, OPV = oral polio vaccine, HBV = hepatitis B vaccine.

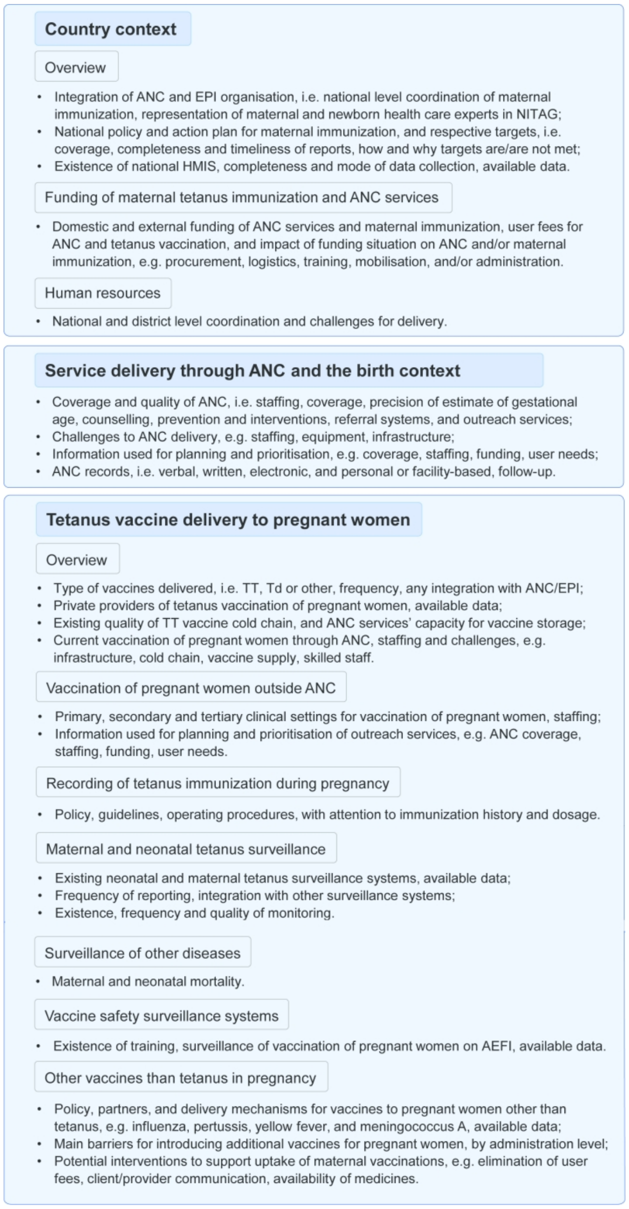

A semi-structured questionnaire will be used to assess the preparedness of antenatal care services for introducing (additional) immunizations for pregnant women in selected low- and lower-middle income countries, and to understand the strengths and weaknesses of current immunization to guide future planning. Internal validation questions are incorporated and probing for further details will be done when deemed necessary by the interviewer(s). Sources of data provided are requested, i.e. if administrative data or personal estimates. ANC = antenatal care, EPI = Expanded Programme on Immunization, NITAG = National Immunisation Technical Advisory Group, HMIS = Health management Information System, TT = tetanus toxoid, Td = tetanus-diphtheria, AEFI = adverse events following immunization.

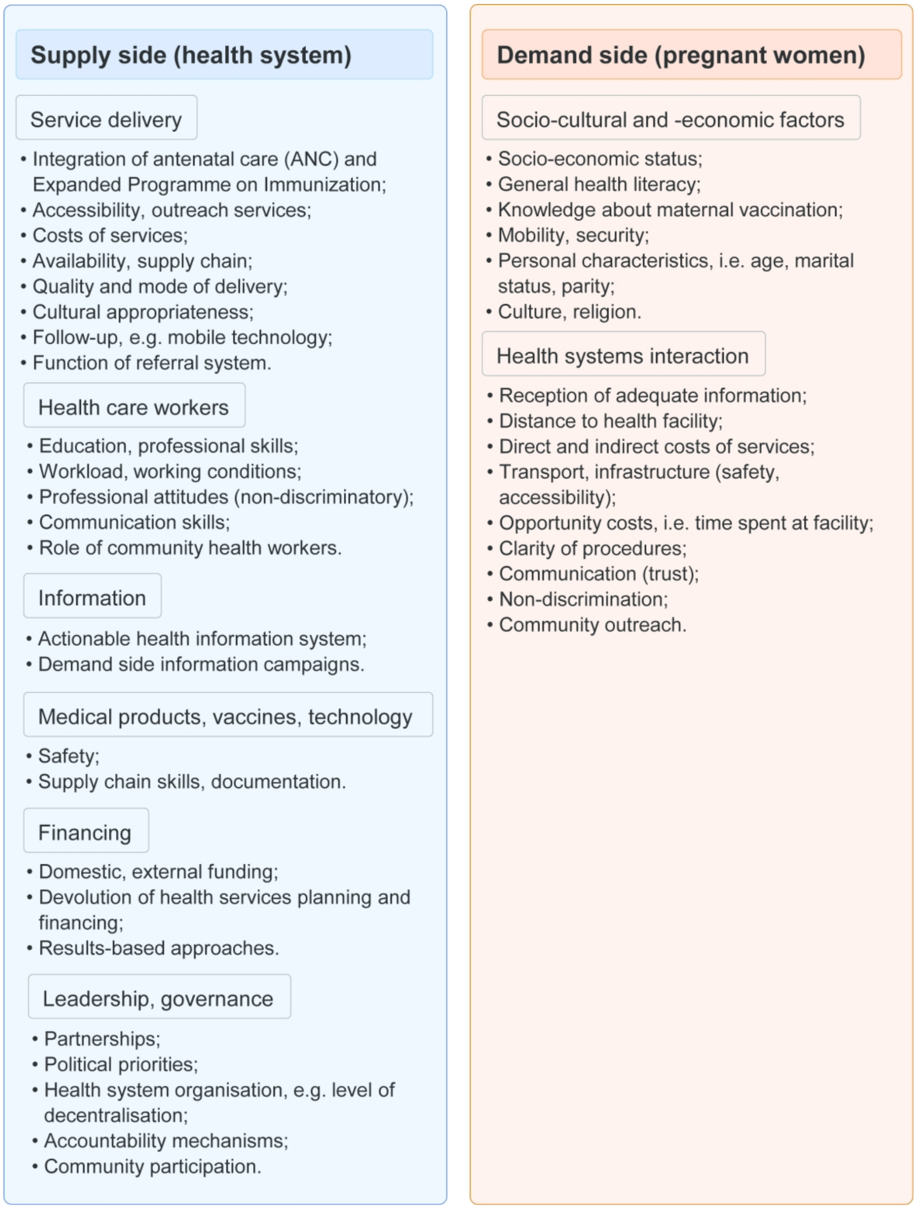

Key informant interviews, health facility visits, and focus group discussions will enable observation and collection of further data on the variables from the previous study phases, in particular at different levels of the health care system, and of socio-cultural and socio-economic factors. End-users, i.e. pregnant women, will not be interviewed as it would require a separate study design, and their perspective will be indirectly included through the participation of community health workers at stakeholder meetings.

BMJ Open

The Maternal Immunization and Antenatal Care Situation Analysis (MIACSA) study protocol: A multi-regional, cross-sectional analysis of maternal immunization delivery strategies to reduce maternal and neonatal morbidity and mortality

Journal:	*BMJ Open*
Manuscript ID	bmjopen-2018-024449.R2
Article Type:	Protocol
Date Submitted by the Author:	08-Feb-2019
Complete List of Authors:	Roos, Nathalie; Organisation mondiale de la Sante, Department of Maternal Newborn Child and Adolescent Health (MCA), Epidemiology Monitoring and Evaluation (EME)<br>Lambach, Philipp; Organisation mondiale de la Sante, Department of Immunization, Vaccines and Biologicals (IVB), Initiative for Vaccine Research (IVR)<br>Mantel, Carsten; MMGH Consulting GmbH; Robert Koch Institute, Department of Infectious Disease Epidemiology<br>Mason, Elizabeth; London School of Hygiene and Tropical Medicine<br>Munoz, Flor M.; Baylor Coll Med, Department of Pediatrics, Section of Infectious Diseases<br>Giles, Michelle; Monash University and Monash Health, Department of Obstetrics and Gynaecology<br>MIACSA, expert advisory panel group<br>Moran, A; World Health Organization, Department of Maternal Newborn Child and Adolescent Health (MCA), Epidemiology Monitoring and Evaluation (EME)<br>Hombach, Joachim; Organisation mondiale de la Sante, Department of Immunization, Vaccines and Biologicals (IVB), Initiative for Vaccine Research (IVR)<br>Diaz, Theresa; World Health Organization, Department of Maternal Newborn Child and Adolescent Health (MCA), Epidemiology Monitoring and Evaluation (EME)
**Primary Subject Heading**:	Global health
Secondary Subject Heading:	Global health, Public health
Keywords:	maternal immunization, maternal mortality, neonatal mortality, study protocol, maternal tetanus, neonatal tetanus

SCHOLARONE™
Manuscripts

For peer review only - http://bmjopen.bmj.com/site/about/guidelines.xhtml

The Maternal Immunization and Antenatal Care Situation Analysis (MIACSA) study protocol: A multi-regional, cross-sectional analysis of maternal immunization delivery strategies to reduce maternal and neonatal morbidity and mortality

Nathalie Roos[1]*, Philipp Lambach[2]*, Carsten Mantel[3], Elizabeth Mason[4], Flor M. Muñoz[5], Michelle L. Giles[6], MIACSA expert advisory panel group**, Allisyn C. Moran[1], Joachim Hombach[2]***, Theresa Diaz[1]***

1 Department of Maternal Newborn Child and Adolescent Health (MCA), Epidemiology Monitoring and Evaluation (EME), World Health Organization (WHO), Geneva, Switzerland

2 Department of Immunization, Vaccines and Biologicals (IVB), Initiative for Vaccine Research (IVR), WHO, Geneva, Switzerland

3 MMGH Consulting GmbH, Zürich, Switzerland and Department of Infectious Disease Epidemiology, Robert Koch Institute, Berlin, Germany

4 London School of Hygiene and Tropical Medicine, London, UK

5 Department of Pediatrics, Section of Infectious Diseases, Baylor College of Medicine, Houston, Texas, USA

6 Department of Obstetrics and Gynaecology, Monash University and Monash Health, Melbourne, VIC, Australia

* First authors

** The MIACSA expert advisory panel (EAP) consists of the following members: Flor M. Muñoz (chair), Michelle L. Giles (co-chair), Mercy Ahun, Martina Baye, Pradeep Haldar, and Matthews Mathai. Observers to EAP meetings included: Carsten Mantel, Elizabeth Mason,

For peer review only - http://bmjopen.bmj.com/site/about/guidelines.xhtml

Sonja Mertens, Jayani Pathirana, Sarah Rendell. Additional WHO experts included: Emily Wootton, Laura Nic Lochlainn, Ahmadu Yakubu, and Sara Rendell.

*** Last authors

Corresponding author: Dr Nathalie Roos, Department of Maternal Newborn Child and Adolescent Health (MCA), Epidemiology Monitoring and Evaluation (EME), WHO, Geneva, Switzerland, email: roosn@who.int, phone: +41 22 791 3191

Word count: 2,603

Key words: maternal immunization; maternal tetanus vaccination, maternal health; neonatal health; maternal tetanus; neonatal tetanus; maternal mortality; neonatal mortality; health care service delivery; antenatal care capacity; study protocol

For peer review only

For peer review only - http://bmjopen.bmj.com/site/about/guidelines.xhtml

ABSTRACT

Introduction. Maternal immunization (MI) is a safe and cost-effective way of preventing neonatal tetanus, and is one of several strategies that aim to reduce the high global rates of maternal and neonatal morbidity and mortality caused by vaccine-preventable diseases. Given the prospect of introducing new maternal vaccines in the near future, it is essential to identify and understand current policies, practices, and unmet needs for introducing and/or scaling up MI in low and middle income countries (LMICs).

Methods and analysis. The Maternal Immunization and Antenatal Care Situation Analysis (MIACSA) is a mixed methods, cross-sectional study that will collect data in four phases: (1) a review of global databases for selected health indicators in 136 LMICs; (2) a structured online survey directed at Maternal, Newborn, and Child Health (MNCH) and Expanded Programme on Immunization (EPI) focal points in all 136 LMICs; (3) semi-structured telephone interviews of 30 selected LMICs; and (4) 10 week-long country visits, including key informant interviews, health facility visits, and focus group discussions. The principal analyses will assess correlations between the various aspects of MI delivery strategies and proxy measures of health systems performance related to vaccine-preventable disease control. The primary outcome will be a typology of existing MI delivery models, and secondary outcomes will include country profiles of child and maternal health indicators, and an MI gaps and needs analysis.

Ethics and dissemination. The protocol was approved by the World Health Organization Ethics Review Committee. The results will be made available in a project report and submitted for publication in peer-reviewed journals that will be shared broadly among global health decision makers, researchers, product developers, and country-level stakeholders.

Registration. Not applicable.

For peer review only - http://bmjopen.bmj.com/site/about/guidelines.xhtml

ARTICLE SUMMARY

Strengths and limitations of this study

- The MIACSA study provides a first time, comprehensive global overview and analysis of existing maternal immunization (MI) delivery strategies in low- and middle-income countries (LMICs).
- The study benefits from a mixed-methods design; a multidisciplinary approach leveraging policy-level, academic, and implementers' experience.
- Limitations include the small number of countries and health care facilities visited within each country included in the study, precluding generalization of country visit findings to a national level.
- End-users' perspective is captured only indirectly through community health workers. Data on maternal immunization service delivery collected through an online survey targeting all low and middle income countries, is analysed within the limitations of the validity of data collected.

For peer review only - http://bmjopen.bmj.com/site/about/guidelines.xhtml

INTRODUCTION

Vaccine-preventable diseases are a major cause of global child morbidity and mortality, particularly in low- and middle-income countries (LMICs).[1] Since the 1990s, public health interventions have more than halved under-five childhood mortality; however, reduction of stillbirths and of neonatal mortality (death in the first 28 days of life) has been slower.[2] This is in part due to the fact that most vaccines cannot be administered to newborns, who, being unable to develop protective responses due to limitations in their immune system, are left particularly vulnerable to infectious diseases. Vaccination of pregnant women, or maternal immunization (MI), has proven to be an effective strategy to reduce neonatal tetanus, and is a potential strategy to reduce the burden of other vaccine-preventable diseases in mothers and infants. Thus, MI is one of several strategies that aim to reach the third sustainable development goal of ending preventable maternal and newborn deaths.[3-5]

Studies have shown that MI can effectively protect the mother, as well as her child, through transplacental transfer of maternal immunoglobulin G (IgG) to the foetus.[6, 7] The Maternal and Neonatal Tetanus Elimination (MNTE) initiative has led the way in the implementation of MI, combining at least two doses of tetanus toxoid containing vaccine (TTCV) during pregnancy (TT2+) with the promotion of hygienic delivery and clean cord care practices, as well as vaccination of children and women of reproductive age, to eliminate maternal and neonatal tetanus as a public health problem. Between the late 1980s and 2015, the MNTE initiative reduced global tetanus-related neonatal mortality by 96%.[8]

TTCV and inactivated influenza vaccines are considered safe and effective for use during pregnancy,[9] and are recommended for pregnant women by the World Health Organization

For peer review only - http://bmjopen.bmj.com/site/about/guidelines.xhtml

(WHO).[6, 10-13] New vaccines, several of which are under development and evaluation, target other important pathogens, such as group B streptococcus (GBS), and respiratory syncytial virus (RSV), and may provide safe and cost-effective protection of mothers and their infants through MI in the future.[14-18]

In order to identify the challenges of implementing current and new vaccines for MI, a better understanding is needed of the capabilities and limitations of existing delivery platforms, such as antenatal care (ANC) services and the Expanded Programme on Immunization (EPI).[19] The capacity of ANC services to deliver vaccines to pregnant women will require thorough assessment, as globally only 62% of women benefit from at least four ANC visits, i.e. the proportion of pregnant women who received 4 or more ANC visits during their last pregnancy (ANC4+), and in Sub-Saharan Africa and South Asia, ANC4+ coverage is only 52% and 46%, respectively.[20] Delivering vaccinations and other essential interventions to women at the necessary timely intervals during pregnancy, as well as documenting the coverage and outcomes of such interventions, requires a robust ANC platform with sufficient personnel and resources.[21]

WHO recommends that pregnant women living in endemic areas are sufficiently immunised against tetanus in order to protect the women and their newborn infants. MI with TTCV is routine in many countries;[22, 23] however, progress of tetanus vaccination in LMICs has faced challenges leading to delays in elimination, and uptake among pregnant women of other vaccines, such as influenza and pertussis vaccines, has been low. As a part of EPI services, routine tetanus immunization during pregnancy has been complemented with supplementary vaccination activities in a majority of countries in order to reach high coverage and achieve

For peer review only - http://bmjopen.bmj.com/site/about/guidelines.xhtml

For peer review only

MNTE goals. A better understanding of MI in the context of both ANC and EPI, including implementation of guidelines and policies, ministerial responsibilities at national and subnational levels, vaccine management including cold chain and logistics, vaccine administration, staff capacity, social mobilisation, vaccine acceptance, and assessment of vaccine safety, may help identify service delivery challenges as well as opportunities to optimise current and future MI efforts.[24]

Closer collaboration between ANC and EPI services could provide a unique and cost-effective opportunity to further strengthen preventive health care measures for women and children under each programme, by reducing missed opportunities for immunization, including MI, as well as reinforcing the delivery of essential health care services.

In view of recent product and policy developments, the WHO, supported by the Bill and Melinda Gates Foundation, aims to identify the knowledge gaps in MI delivery strategies by mapping the strengths and challenges of existing ANC and immunization services for pregnant women in LMICs through the Maternal Immunization and Antenatal Care Situation Analysis (MIACSA) project. The results will provide the evidence for a typology of MI delivery models, as well as identify the capacity needs and key system changes required to introduce new maternal vaccines and/or strengthen vaccine delivery for MI in LMICs. Ultimately, the project aims to identify and understand current MI and ANC related policies, practices and the need for strengthening both immunization and maternal child health care services, and how they could accommodate new MI vaccines.

For peer review only - http://bmjopen.bmj.com/site/about/guidelines.xhtml

METHODS AND ANALYSIS

Patient and Public Involvement.

The development of the research questions were influenced by an interdisciplinary group of international experts for the MIACSA project. The project did not include patients, but restricted itself to national level program managers and health facilities where health workers responded to interviews in their professional capacity.

Study design and data collection

Between November 2016 and December 2018, a mixed-methods, cross-sectional study will be carried out in four phases to assess key health system features related to the implementation of MI (Figure 1). An expert advisory panel (EAP) consisting of specialists in immunization, maternal and neonatal health, MI implementation and social sciences, will provide technical advice on the study design, the development of research questions and surveys, the data collection methods, and the results interpretation. In addition to following WHO standards for global monitoring surveys, all data collection tools and standard operating procedures will be reviewed and endorsed by the EAP. The surveys and country visits will be conducted in local languages when needed.

Desk review of global data (Data collection phase 1). The first phase will consist of collecting key health indicators of LMICs to create outlines of country profiles, focusing on ANC and EPI services. A desk review of pre-defined health indicators (Figure 2) from 136 LMICs will be conducted from existing global data sources, including Demographic and Health Surveys (DHS)/Multiple Indicator Cluster Surveys (MICS), WHO/United Nations Children's Fund

For peer review only

For peer review only - http://bmjopen.bmj.com/site/about/guidelines.xhtml

(UNICEF) estimates of national immunization coverage, WHO/UNICEF Joint Reporting Forms (JRF), MNTE reports, and WHO Maternal, Newborn, Child, and Adolescent Health (MNCAH) policy survey. The indicators will focus on governance and policy environment, health systems performance, and immunization activities, including MI. Data from phase 1 will be compiled in a database for analysis of the study's research questions, and will inform the selection of countries for phase 3 (see below).

Global online survey (Data collection phase 2). The country profiles established in phase 1 will be completed and, if needed, updated by an online survey with WHO Regional Offices, national Ministry of Health (MoH) focal points from Maternal, Newborn and Child Health (MNCH) and EPI programmes and their WHO Country Office counterparts in all LMICs, using a structured questionnaire (Figure 3). Data will be collected on service delivery models of maternal tetanus vaccination, including delivery platforms, programme funding, disease surveillance, and vaccine safety surveillance. Data on maternal vaccines other than tetanus will be included when pertinent. A draft questionnaire will be piloted in advance of the survey. Non-responders will be followed-up by telephone and email. Revisions following queries on missing, erroneous, or inconsistent data will be done at country level.

Telephone interviews (Data collection phase 3). In order to understand how existing health care delivery services could be adapted to implement MI beyond tetanus immunization, further data will be collected on delivery platforms for maternal tetanus vaccination in LMICs. In-depth telephone interviews will be conducted with EPI and MNCH programme officers responsible for MI at the MoH in a sample of 30 countries, using a semi-structured questionnaire (Figure 4). The countries will be selected based on performance of MI as

For peer review only - http://bmjopen.bmj.com/site/about/guidelines.xhtml

assessed by coverage of maternal TTCV and ANC, geographic representation, and recommendations from WHO Regional Offices on MI priorities. The countries will be stratified into four groups; high and low maternal tetanus vaccination performance measured as protection at birth (PAB), i.e. the proportion of newborns protected at birth against neonatal tetanus, with a cut-off of 90%, and high and low ANC performance (with a cut-off of the median ANC4+ coverage in countries with available data). PAB was identified as a more reliable proxy measure than TT2+, as the issue of not including already vaccinated women in the numerator used for estimating the latter indicator would be avoided. The PAB cut-off level was set based on the target required to attain and sustain MNTE, whereby >80% of pregnant women are immunized against tetanus. The country selection will include a representation of all MI delivery models and WHO regions, with a focus on Africa and South-East Asia where maternal and neonatal mortality are highest, and will ensure inclusion of high performing countries in order to include likely early adopters of new maternal vaccines and learning cases of best practices.

The interviews will collect data on the policy, governance, and funding environment for EPI and ANC programmes, ANC delivery, and maternal tetanus vaccination including monitoring and evaluation of results. The questionnaire will be shared with WHO country office focal points and MoH MNCH and EPI managers for compilation in advance of the teleconference, allowing for discussion and clarification when needed during the actual interview. Responses will be recorded using standard data entry procedures, and may be voice-recorded if consent is obtained by the interviewees. Any discordant responses will be attempted to be resolved by consensus, and incomplete responses will be followed up. A summary of key findings will be shared with the participants to confirm the responses were correctly captured.

For peer review only - http://bmjopen.bmj.com/site/about/guidelines.xhtml

Country visits (data collection phase 4). Finally, in-country visits will be conducted in order to collect data on MI from key decision-makers and implementers at every level of the health care system, as well as to determine actual delivery, capacity and coordination of ANC and EPI services, on both supply and demand sides of the health care services (Figure 5). Ten countries will be selected based on high, medium, or low performance of MI systems as assessed by PAB and TT2+, a range of different MI delivery models (e.g. degree of coordination between EPI and ANC in MI delivery), and agreement by senior national and subnational MNCH and EPI staff for study visits. The final country selection will ensure representation of the range of MI delivery models, and will include high-performing countries, MNTE priority countries, and countries with high ANC4+ coverage. Site visits will include ANC and EPI sites and session observations, focus group discussions, and in-depth interviews. The week-long visits will be piloted in two countries to adjust and refine the data collection tools and the standard operating procedures, and data from these two countries will be included in the final analysis.

An initial joint focus group discussion will be held with national-level stakeholders, followed by key informant interviews with stakeholders pertinent to MI, ANC, and EPI services at subnational levels of the health care system, including decision and policy makers, technical and financial parties, and civil society, such as non-governmental organisations. The study will aim to conduct a total of twelve health facility visits taking into account a balance of geographical locations, urban and rural areas, and - if possible - different types of health facilities (e.g. small and larger health units). The country visits will be concluded with an on-site debriefing and joint data analysis with MoH MNCH and EPI focal points and other main

For peer review only - http://bmjopen.bmj.com/site/about/guidelines.xhtml

country-level stakeholders. End-users, i.e. pregnant women, will not be interviewed as it would require a separate study design; however, their perspective will be indirectly included through the participation of community health workers at stakeholder meetings.

Data analysis plan

The cross-sectional data analyses will be carried out over four data collection 4 phases (desk review of global data, online questionnaire and indepth country interviews, and country visits). The first three will yield quantitative data. The last two data collection phases will also provide an in-depth qualitative analyses of data collected from a select number of countries. Below we describe the analyses for each phase.

Desk review of global data (phase 1)

The MIACSA project will conduct a desk review of global databases (Joint reporting form (JRF), United Nations (UN) mortality reports, Demographic health survey (DHS), Multiple Cluster Indicators Survey (MICS), WHO MNCAH policy survey database, maternal and neonatal tetanus elimination (MNTE) database, WHO/UNICEF Estimates of National Immunization Coverage (WUENIC)) targeting 136 low- and middle income countries (LMIC).

The primary outcome variable (dependant variable) to asses MI performance will be PAB (cut-off level <90% and ≥90%) and the independent variables will include country economic level, immunization coverage, mortality, service coverage, available ANC and vaccination policies and availability of a national immunization advisory committee (Figure 2).

We will first asses the database for completeness of data. We will also conduct a sensitivity analysis based on imputation of data based on available predictors for countries with missing data on PAB. Results from the complete case analysis will be compared with the sensitivity

For peer review only - http://bmjopen.bmj.com/site/about/guidelines.xhtml

analysis to explore bias due to missing data.

We will conduct bivariate analyses to assess whether the dependant variables are associated with the independent variables. We will also do multivariable analyses within subgroups, since vaccinations may differ by other factors (e.g., WHO Regions; GAVI status; World Bank income level; MNTE; female literacy rate).

For continuous variables we will first assess the normality using the Shapiro-Wilk test. If needed, we will make appropriate transformations to normalize the data or group them into categories as necessary. We will then compare the distributions of the variables by groups with two-sided chi-square (categorical variables) or t-tests (continuous variables) or the equivalent non-parametric tests (e.g., Fisher's exact or Wilcoxon/Kruskal-Wallis), as appropriate. A two-sided p-value of 0.05 will be considered as significant.

To create a multivariable model, we will include all variables that are significantly associated with the dependant variable and those variables which have shown association within the available literature. We will then asses for collinearity and remove one of the variables if collinearity is found. We will also assess for interactions and will create interaction terms to be included in the model if any interactions are found. Both forward and backward elimination will be conducted to assess the goodness of fit and create the final model.

Global online survey (phase 2)

The variables are based on the online survey as described above. For a summary of the included components see Figure 3. Data from the online survey will be checked for completeness and consistency and coded to reflect skip patterns. The complete data set for analyses will include PAB from phase 1 (desk review database) and will be linked with the database containing responses for the global online questionnaire. Descriptive analyses will

For peer review only

be conducted including summary measures. Bivariate analyses will be conducted to assess the associations between the questionnaire variables and the dependant variable PAB and the significance of the relationship will be tested with Fisher's Exact test. Logistic regression models will be used to assess the relationship between the responses in the online questionnaire and high coverage of PAB >=90% independently. These models will be created as described in phase 1. A two-sided p-value of 0.05 will be considered as significant.

Telephone conferences (quantitative analyses, phase 3)

The primary objective is to provide descriptive information about maternal immunization services and its organization (Figure 4).

Data from this phase will be checked for completeness and consistency and coded to reflect skip patterns. Descriptive analyses will be conducted including summary measures.

Qualitative analyses based on country visits and telephone conferences (phase 4)

Ten countries will be visited to conduct qualitative interviews at national and subnational level. Resulting qualitative information and from these visits as well as from telephone interview conducted in the previous phase will be used in a thematic analysis applied by trained qualitative data analysts to the following qualitative data sources: comments and free-text responses to telephone interview questions (phase 3), semi-structured interviews with community health workers (phase 4), comments and free-text response to stakeholder and facility manager interviews (phase 4), comments provided during debrief sessions with national level stakeholders in-country (phase 4). Thematic analysis will be applied to intra-case and cross-case analysis. First, an intra-case analysis will organize and reduce qualitative findings within each country along two criteria: (1) relevance of finding to research questions and (2) relative frequency of finding across data sources. Second, a cross-case analysis will

For peer review only - http://bmjopen.bmj.com/site/about/guidelines.xhtml

organize findings across countries into themes generated from research questions and sub-themes generated from grounded analysis of data collected. Two qualitative data analysts will co-organize and reduce intra-country findings. For cross-country findings, qualitative analysts will independently generate themes and sub-themes for cross-case analysis and will then resolve any inter-coder divergence in themes and sub-themes based on relevance of theme to data source, relevance of theme to research questions and robustness of theme relative to alternative themes. See figure 4 and 5 for the included components.

Consolidated data analysis

To inform the development of a typology of MI delivery models approaches in LMICs quantitative and qualitative data analysis results will be consolidated in a global analysis of MI and ANC service delivery approaches in countries as well as individual country profiles that shall support countries to conduct self-assessments of their MI and ANC systems strengths and capacity gaps. Based on the advice of the project's advisory group, a checklist approach will be considered to provide a profiling for countries with sufficient data available, including indicators on policy and governance, financing, programme management, service delivery systems and demand side issues. Ultimately, such a profiling shall help countries and other MI stakeholders to identify the needs for targeted support to strengthen existing MI programmes or to reach readiness to introduce future programmes.

Limitations

The data analyses will take into account the limitations of the study, including the reliability of the selected outcome measures, i.e. PAB, potential biases introduced by the limited number of countries for which in-depth information will be available, i.e. through telephone interviews and in-country visits, selective sampling of in-country site visit locations, missing

For peer review only - http://bmjopen.bmj.com/site/about/guidelines.xhtml

307 data, and the fact that the end-users' perspective will be captured only indirectly through
308 facility and community based health workers.

For peer review only

For peer review only - http://bmjopen.bmj.com/site/about/guidelines.xhtml

ETHICS AND DISSEMINATION

Ethical considerations. The first three phases of the study are exempt from ethical permission as participants will provide information on operations and administration of public health services on a purely professional basis, and without disclosure of person-identifiable information. The protocol for the country visits was approved by the WHO Research Ethics Review Committee (ERC.0002908).

Country ownership will be ensured through transfer of responsibility for the data provision to in-country focal points, and by joint, on-site analysis of the data collected during the country visits with the main stakeholders. The study aims to contribute to the evidence needed to ensure more equitable access to high-impact global health interventions, such as MI.[25]

Data management and dissemination

The data will be managed and analysed by data clerks who were not part of the data collection. Anonymised data from surveys and key informant interviews, excluding any confidential information as identified by the in-country focal points, will be uploaded to a publicly available data repository hosted by the WHO. Recordings from country interviews will be transcribed before the qualitative analyses and destroyed at completion of the data analyses.

The results will be submitted for publication in peer-reviewed journals, as well as in a MIACSA project report that will be shared widely with global health decision makers, researchers, product developers, and implementers. The report and/or specific aspects of the

For peer review only - http://bmjopen.bmj.com/site/about/guidelines.xhtml
For peer review only

project, will be presented at international stakeholder meetings, with the ultimate aim to establish a knowledge network of countries exploring MI implementation strategies. Further, the results will be shared through summaries on the WHO website and in public fora.

To ensure wide distribution of the project findings to the international scientific community and national stakeholders involved in maternal immunization, findings will be also shared at the end of the project through a large stakeholder convening. At this meeting, key aspects of maternal tetanus vaccination service delivery mechanisms and antenatal care capacities identified in select countries will be discussed to enable exchange of lessons learnt between select participating countries and to discuss generalizable lessons learnt that may improve maternal immunization service delivery through an integrated platform considering Immunization and Maternal Child Health Care mechanisms.

Dissemination of the MIACSA results will aim to provide advice on best practices, policy requirements, capacity needs, and health system changes needed for successful introduction and integration of new maternal vaccines into national health systems, including ANC and EPI services, in LMICs.

For peer review only

For peer review only - http://bmjopen.bmj.com/site/about/guidelines.xhtml

REFERENCES

1. UNICEF WHO, and World Bank Group. Levels and Trends in Child Mortality: Report 2017. New York, 2017.

2. Lawn JE, Blencowe H, Oza S, et al. Every Newborn: progress, priorities, and potential beyond survival. *Lancet* 2014;384:189-205.

3. Sobanjo-Ter Meulen A, Abramson J, Mason E, et al. Path to impact: A report from the Bill and Melinda Gates Foundation convening on maternal immunization in resource-limited settings; Berlin - January 29-30, 2015. *Vaccine* 2015;33:6388-95.

4. You D, Hug L, Ejdemyr S, et al. Global, regional, and national levels and trends in under-5 mortality between 1990 and 2015, with scenario-based projections to 2030: a systematic analysis by the UN Inter-agency Group for Child Mortality Estimation. *Lancet* 2015;386:2275-86.

5. WHO. Trends in maternal mortality: 1990 to 2015. Estimates by WHO, UNICEF, UNFPA, World Bank Group and the United Nations Population Division. Geneva: World Health Organization, 2015.

6. Englund JA, Mbawuike IN, Hammill H, et al. Maternal immunization with influenza or tetanus toxoid vaccine for passive antibody protection in young infants. *J Infect Dis* 1993;168:647-56.

7. Puck JM, Glezen WP, Frank AL, et al. Protection of infants from infection with influenza A virus by transplacentally acquired antibody. *J Infect Dis* 1980;142:844-9.

8. WHO. http://www.who.int/immunization/diseases/MNTE_initiative/en/ Accessed 22 January 2018.

9. Keller-Stanislawski B, Englund JA, Kang G, et al. Safety of immunization during pregnancy: a review of the evidence of selected inactivated and live attenuated vaccines. *Vaccine* 2014;32:7057-64.

For peer review only - http://bmjopen.bmj.com/site/about/guidelines.xhtml

373 10. WHO. Safety of immunization during pregnancy: A review of the evidence. World Health Organization. Geneva, 2014.
375 11. Hviid A, Svanstrom H, Molgaard-Nielsen D, et al. Association Between Pandemic Influenza A(H1N1) Vaccination in Pregnancy and Early Childhood Morbidity in Offspring. *JAMA Pediatr* 2017;171:239-48.
378 12. Vaccines against influenza WHO position paper - November 2012. *Wkly Epidemiol Rec* 2012;87:461-76.
380 13. Eick AA, Uyeki TM, Klimov A, et al. Maternal influenza vaccination and effect on influenza virus infection in young infants. *Arch Pediatr Adolesc Med* 2011;165:104-11.
383 14. Russell LB, Kim SY, Cosgriff B, et al. Cost-effectiveness of maternal GBS immunization in low-income sub-Saharan Africa. *Vaccine* 2017;35:6905-14.
385 15. Donders GG, Halperin SA, Devlieger R, et al. Maternal Immunization With an Investigational Trivalent Group B Streptococcal Vaccine: A Randomized Controlled Trial. *Obstet Gynecol* 2016;127:213-21.
388 16. Chaithongwongwatthana S, Yamasmit W, Limpongsanurak S, et al. Pneumococcal vaccination during pregnancy for preventing infant infection. *Cochrane Database Syst Rev* 2015;1:CD004903.
391 17. PATH. Advancing maternal immunization (AMI) http://sites.path.org/cvia/our-disease-targets/respiratory-syncytial-virus/advancing-maternal-immunization-ami/2017 Accessed 22 January 2018.
394 18. Glenn GM, Smith G, Fries L, et al. Safety and immunogenicity of a Sf9 insect cell-derived respiratory syncytial virus fusion protein nanoparticle vaccine. *Vaccine* 2013;31:524-32.

For peer review only - http://bmjopen.bmj.com/site/about/guidelines.xhtml

19. Singh K, Story WT, Moran AC. Assessing the Continuum of Care Pathway for Maternal Health in South Asia and Sub-Saharan Africa. *Matern Child Health J* 2016;20:281-9.

20. UNICEF. Antenatal care coverage: At least four visits https://data.unicef.org/topic/maternalhealth/antenatal-care/#2017 Accessed 10 April 2017.

21. Hodgins S, D'Agostino A. The quality-coverage gap in antenatal care: toward better measurement of effective coverage. *Glob Health Sci Pract* 2014;2:173-81.

22. Pecenka C, Munthali S, Chunga P, et al. Maternal influenza immunization in Malawi: Piloting a maternal influenza immunization program costing tool by examining a prospective program. *PLoS One* 2017;12:e0190006.

23. McMorrow ML, Emukule GO, Obor D, et al. Maternal influenza vaccine strategies in Kenya: Which approach would have the greatest impact on disease burden in pregnant women and young infants? *PLoS One* 2017;12:e0189623.

24. Nesin M, Read J, Koso-Thomas M, et al. Maternal Immunization: Current status and future prospects. *Vaccine* 2015;33:6371.

25. Verweij M, Lambach P, Ortiz JR, et al. Maternal immunisation: ethical issues. *Lancet Infect Dis* 2016;16:e310-e14.

For peer review only - http://bmjopen.bmj.com/site/about/guidelines.xhtml

AUTHORS' CONTRIBUTIONS

NR and PL designed the study with inputs from TD and JH; CM, EM, FMM, MLG, TD, JH, AM and the MIACSA expert advisory panel group drafted the protocol with NR and PL; and all authors reviewed and approved the final manuscript version.

ACKNOWLEDGMENTS

The investigators wish to thank the EPI and MNCH focal points at the regional and national WHO offices for their helpful contributions to the planning of the study, and the external specialists of the Expert Advisory Panel for their valuable advice and guidance on development of the protocol methodology. The authors also wish to thank Dr. Peter Mark Jourdan for assistance in writing and editing the manuscript. There was no involvement from the public or patients in the project development.

FUNDING

This work was supported by the Bill and Melinda Gates Foundation, grant number OPP1156378.

COMPETING INTERESTS

The authors declare no competing interests.

For peer review only - http://bmjopen.bmj.com/site/about/guidelines.xhtml

FIGURE LEGENDS

Figure 1. Key health system features studied by the MIACSA project.

EPI = Expanded Programme on Immunization, ANC = antenatal care, AEFI = adverse events following immunization, TT = tetanus toxoid.

Figure 2. Study phase 1: List of indicators for the review of global databases.

CES = coverage evaluation survey, WUENIC = World Health Organization (WHO)/United Nations Children's Fund (UNICEF) estimates of national immunization coverage, BCG = Bacillus Calmette-Guérin vaccine, DPT1 = first dose of diphtheria-pertussis-tetanus vaccine, DPT3 = third dose of diphtheria-pertussis-tetanus vaccine, HepB1 = first dose of hepatitis B vaccine, HepB3 = third dose of hepatitis B vaccine, Hib1 = first dose of *Haemophilus influenzae* type B vaccine, Hib3 = third dose of *H. influenzae* type B vaccine, MCV1 = first dose of measles-containing vaccine, MCV2 = second dose of measles-containing vaccine, PcV1 = first dose of pneumococcal conjugate vaccine, PcV3 = third dose of pneumococcal conjugate vaccine, Pol1 = first dose of polio-containing vaccine, Pol3 = third dose of polio-containing vaccine, TT1 = first dose of tetanus toxoid vaccine, TT1+ = at least one dose of tetanus toxoid vaccine, TT2 = second dose of tetanus toxoid vaccine, TT2+ = at least two doses of tetanus toxoid vaccine, TT3 = third dose of tetanus toxoid vaccine, TT4 = fourth dose of tetanus toxoid vaccine, TT5 = fifth dose of tetanus toxoid vaccine, RCV1 = first dose of rubella-containing vaccine, RotaC = second or third dose of rotavirus vaccine depending on number of doses recommended in national schedule, YFV = yellow fever vaccine.

For peer review only

For peer review only - http://bmjopen.bmj.com/site/about/guidelines.xhtml

Figure 3. Study phase 2: Variables collected from online survey of 136 LMICs.

A structured questionnaire will be used to determine which service delivery platforms are in place for tetanus vaccination of pregnant women in low- and middle-income countries (LMICs), and to understand how existing health services could be adapted to implement maternal immunization beyond tetanus vaccination. Internal validation questions are incorporated in the questionnaire, and sources of data are requested, i.e. if administrative data or personal estimates. ANC = antenatal care, EPI = Expanded Programme on Immunization, TT = tetanus toxoid, Td = tetanus-diphtheria, Tdap = tetanus-diphtheria-acellular pertussis, AEFI = adverse events following immunization, TT2+ = at least 2 doses of tetanus toxoid vaccine during pregnancy, PAB = protection at birth, BCG = Bacillus Calmette-Guérin vaccine, OPV = oral polio vaccine, HBV = hepatitis B vaccine.

Figure 4. Study phase 3: Variables collected from interviews of 30 selected LMICs.

A semi-structured questionnaire will be used to assess the preparedness of antenatal care services for introducing (additional) immunizations for pregnant women in selected low- and lower-middle income countries, and to understand the strengths and weaknesses of current immunization to guide future planning. Internal validation questions are incorporated and probing for further details will be done when deemed necessary by the interviewer(s). Sources of data provided are requested, i.e. if administrative data or personal estimates. ANC = antenatal care, EPI = Expanded Programme on Immunization, NITAG = National Immunisation Technical Advisory Group, HMIS = Health management Information System, TT = tetanus toxoid, Td = tetanus-diphtheria, AEFI = adverse events following immunization.

For peer review only - http://bmjopen.bmj.com/site/about/guidelines.xhtml

Figure 5. Study phase 4: Country study analysis framework for 10 country visits.

Key informant interviews, health facility visits, and focus group discussions will enable observation and collection of further data on the variables from the previous study phases, in particular at different levels of the health care system, and of socio-cultural and socio-economic factors. End-users, i.e. pregnant women, will not be interviewed as it would require a separate study design, and their perspective will be indirectly included through the participation of community health workers at stakeholder meetings.

For peer review only

For peer review only - http://bmjopen.bmj.com/site/about/guidelines.xhtml

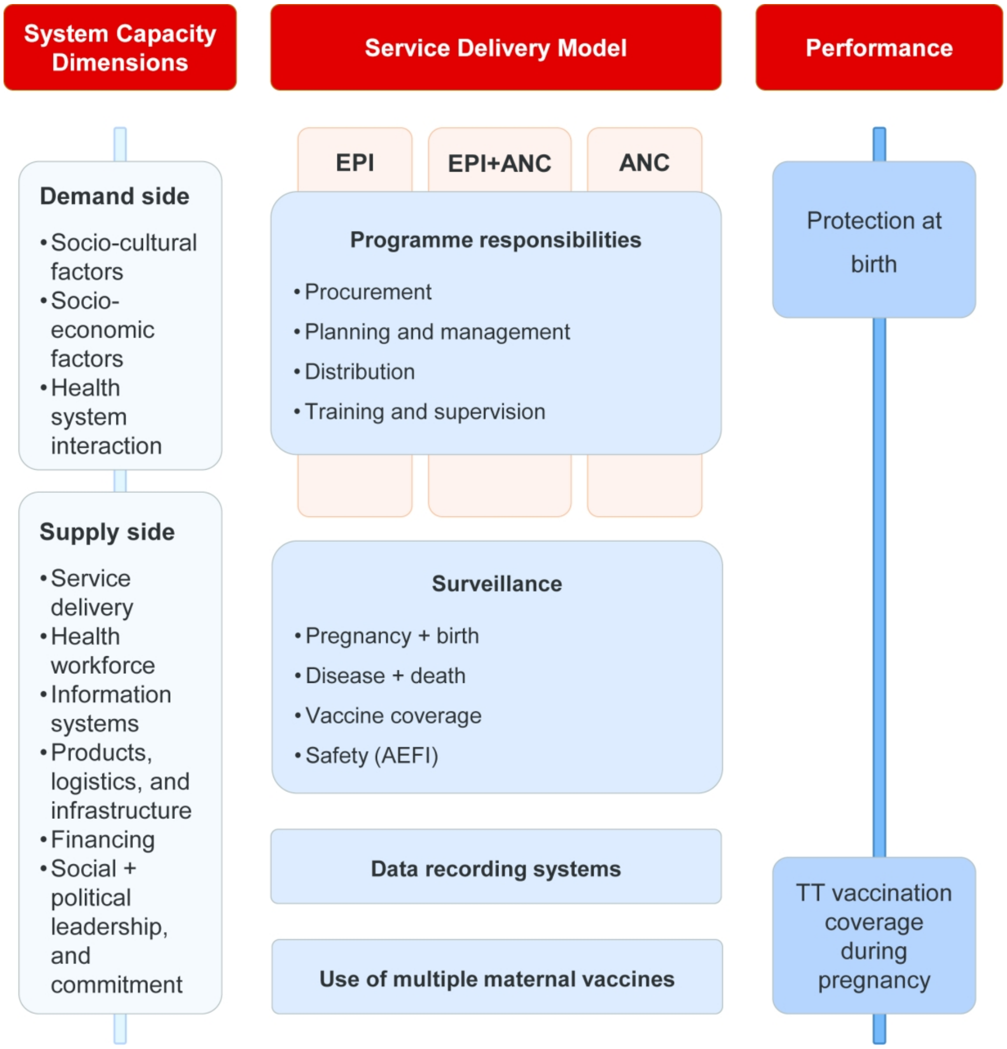

EPI = Expanded Programme on Immunization, ANC = antenatal care, AEFI = adverse events following immunization, TT = tetanus toxoid.

General

- World Bank income classification;
- Female literacy rate.

Health systems

General

- Health systems classification;
- Birth cohort (most recent year of available data);
- Target population of pregnant women.

Governance and policy environment

- Percentage of total expenditure on routine immunization financed by government funds;
- Existence of national immunization technical advisory group (NITAG);
- National policy on minimum antenatal care (ANC) visits;
- Eligibility for global vaccine alliance (GAVI) support.

Health systems performance

- Maternal, neonatal, and infant mortality;
- Stillbirth rates;
- Physician and midwife densities;
- Institutional deliveries;
- Coverage of a minimum of four ANC visits (ANC4+).

Vaccination, including maternal immunization

- Number of confirmed tetanus and neonatal cases;
- Coverage of at least 2 doses of TT vaccine during pregnancy (TT2+);
- Tetanus toxoid (TT) vaccine as a proportion of CES and WUENIC vaccines, i.e. BCG, DPT1, DPT3, HepB1, HepB3, Hib1, Hib3, MCV1, MCV2, PAB, PcV1, PcV3, Pol1, Pol3, TT1, TT1+, TT2, TT2+, TT3 TT4, TT5, RCV1, RotaC, YFV;
- Proportion protected at birth from neonatal tetanus; i.e. protection at birth (PAB);
- TT containing vaccine(s) administered to pregnant women during routine visits;
- Most recent TT supplementary immunization activities (SIA), age range and size of target population, vaccination coverage, vaccine presentation, and year of next planned activity;
- Number of adverse events following immunization (AEFI);
- Maternal and Neonatal Tetanus Elimination (MNTE) status (year of elimination);
- Influenza vaccine administered to pregnant women;
- Pertussis vaccine administered to pregnant women.

Immunization-associated activities

- Vitamin A supplementation.

Figure 2. Study phase 1: List of indicators for the review of global databases. CES = coverage evaluation survey, WUENIC = World Health Organization (WHO)/United Nations Children's Fund (UNICEF) estimates of national immunization coverage, BCG = Bacillus Calmette-Guérin vaccine, DPT1 = first dose of diphtheria-pertussis-tetanus vaccine, DPT3 = third dose of diphtheria-pertussis-tetanus vaccine, HepB1 = first dose of hepatitis B vaccine, HepB3 = third dose of hepatitis B vaccine, Hib1 = first dose of Haemophilus influenzae type B vaccine, Hib3 = third dose of H. influenzae type B vaccine, MCV1 = first dose of measles-containing vaccine, MCV2 = second dose of measles-containing vaccine, PcV1 = first dose of pneumococcal conjugate vaccine, PcV3 = third dose of pneumococcal conjugate vaccine, Pol1 = first dose of polio-containing vaccine, Pol3 = third dose of polio-containing vaccine, TT1 = first dose of tetanus toxoid vaccine, TT1+ = at least one dose of tetanus toxoid vaccine, TT2 = second dose of tetanus toxoid vaccine, TT2+ = at least two doses of tetanus toxoid vaccine, TT3 = third dose of tetanus toxoid vaccine, TT4 = fourth dose of tetanus toxoid vaccine, TT5 = fifth dose of tetanus toxoid vaccine, RCV1 = first dose of rubella-containing vaccine, RotaC = second or third dose of rotavirus vaccine depending on number of doses recommended in national schedule, YFV = yellow fever vaccine.

Service delivery models

Routine maternal tetanus vaccination

- Policy content and coverage data;
- Existing delivery models, e.g. facility-based ANC and EPI/immunization services, outreach services, and regular and ad hoc health campaigns;
- Type(s) of vaccines administered, i.e. TT, Td, Tdap (adult formulation).

Integrated health campaigns for maternal tetanus vaccination

- Programme management and coverage data;
- Existing campaigns integrated with vaccination, e.g. deworming, vitamin A, malaria, nutrition;
- Past and future schedules of integrated health campaigns.

EPI, ANC or other organisation of maternal tetanus vaccination

- National level coordination planning and management;
- Training (rationale, safety and AEFI surveillance) and supervision of vaccinators;
- Vaccine procurement and distribution;
- Monitoring and evaluation, i.e. records (ANC or EPI-based personal, clinic, or electronic), frequency of performance assessment, monitoring indicators, e.g. TT2+, PAB.

Funding for maternal tetanus vaccination programme

- Domestic and external funding.

Disease surveillance

- Maternal and neonatal tetanus, i.e. passive, sentinel, active, community-based;
- Other health indicators, i.e. congenital rubella syndrome, neonatal sepsis, neonatal mortality, maternal mortality, BCG at birth, OPV at birth, HBV at birth, other.

ANC capacity for maternal immunization

- Policy for ANC, i.e. number of visits, settings for ANC provision, i.e. government or private health facility/hospital, clinic, outreach programme;
- Any user fees for ANC and maternal immunization.

Vaccine safety surveillance

- Surveillance of AEFI following immunization in general and maternal immunization;
- Any available surveillance data.

Other maternal vaccines

- Routine maternal immunization, e.g. influenza, pertussis, or other;
- Programme management, i.e. EPI, ANC, or other responsible for planning, training, supervision, procurement, and distribution.

A structured questionnaire will be used to determine which service delivery platforms are in place for tetanus vaccination of pregnant women in low- and middle-income countries (LMICs), and to understand how existing health services could be adapted to implement maternal immunization beyond tetanus vaccination. Internal validation questions are incorporated in the questionnaire, and sources of data are requested, i.e. if administrative data or personal estimates. ANC = antenatal care, EPI = Expanded Programme on Immunization, TT = tetanus toxoid, Td = tetanus-diphtheria, Tdap = tetanus-diphtheria-acellular pertussis, AEFI = adverse events following immunization, TT2+ = at least 2 doses of tetanus toxoid vaccine during pregnancy, PAB = protection at birth, BCG = Bacillus Calmette-Guérin vaccine, OPV = oral polio vaccine, HBV = hepatitis B vaccine.

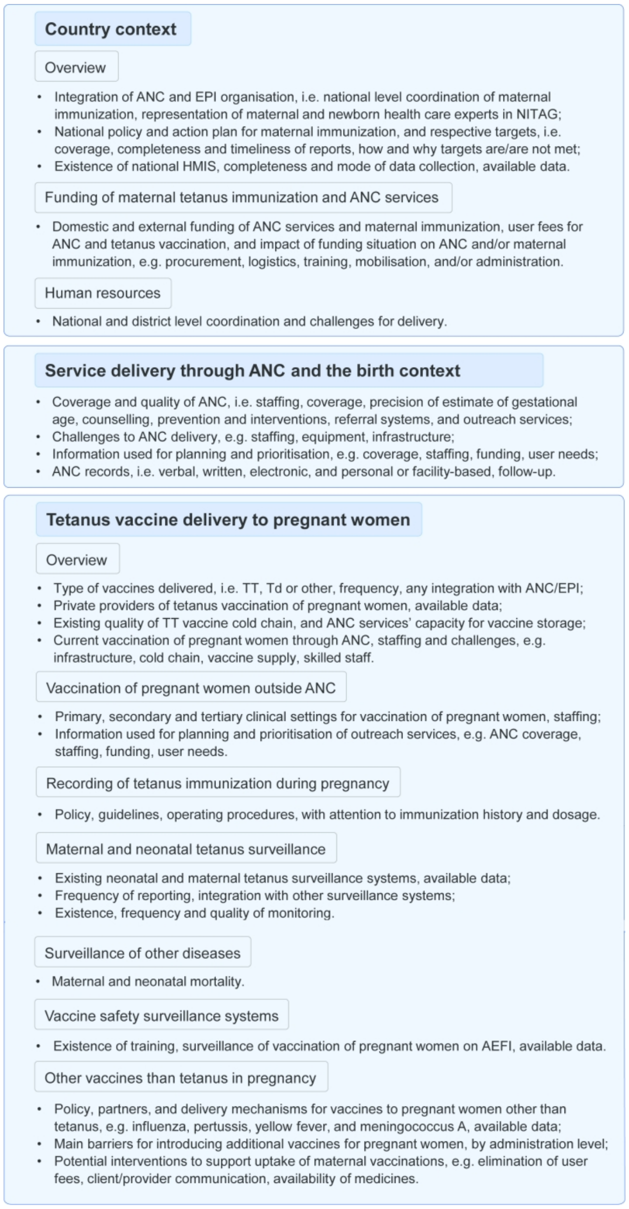

A semi-structured questionnaire will be used to assess the preparedness of antenatal care services for introducing (additional) immunizations for pregnant women in selected low- and lower-middle income countries, and to understand the strengths and weaknesses of current immunization to guide future planning. Internal validation questions are incorporated and probing for further details will be done when deemed necessary by the interviewer(s). Sources of data provided are requested, i.e. if administrative data or personal estimates. ANC = antenatal care, EPI = Expanded Programme on Immunization, NITAG = National Immunisation Technical Advisory Group, HMIS = Health management Information System, TT = tetanus toxoid, Td = tetanus-diphtheria, AEFI = adverse events following immunization.

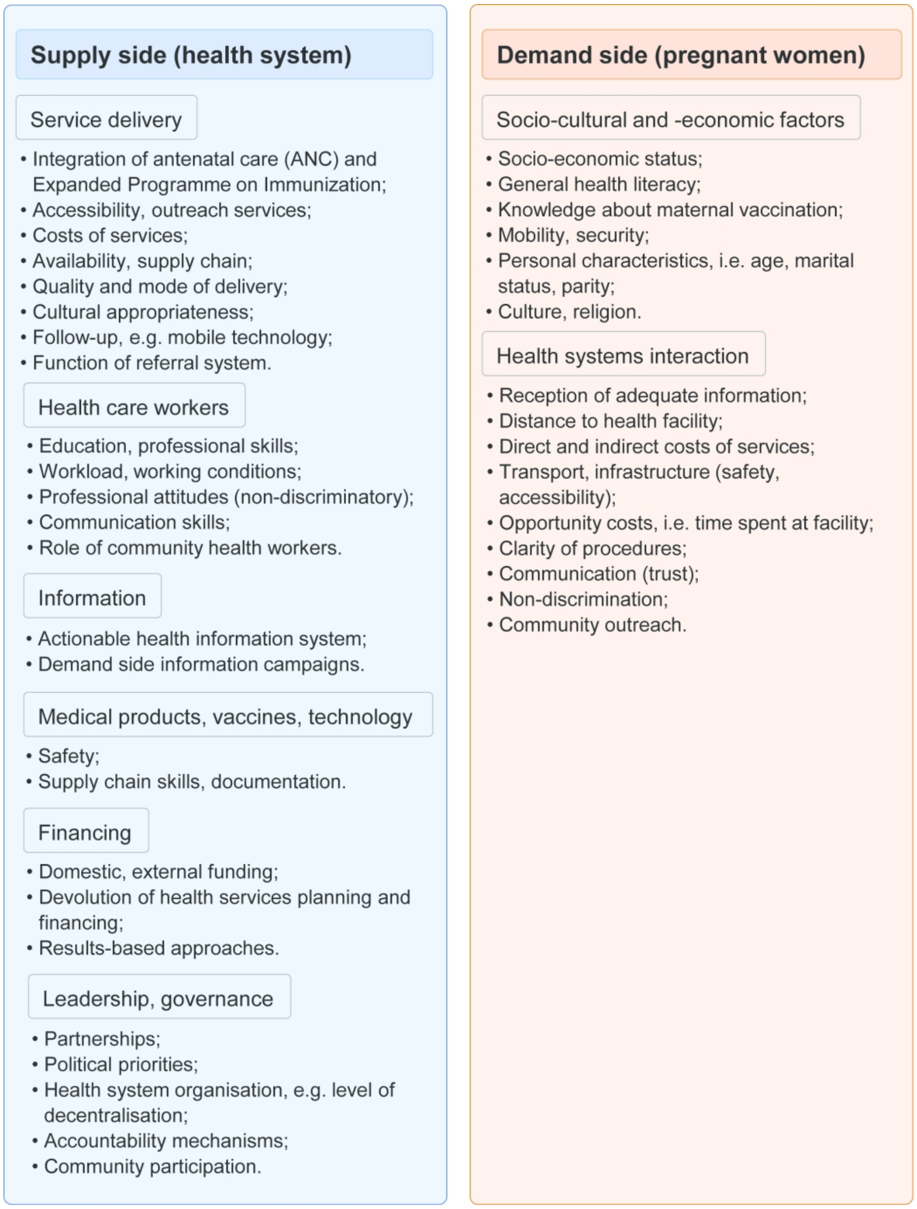

Key informant interviews, health facility visits, and focus group discussions will enable observation and collection of further data on the variables from the previous study phases, in particular at different levels of the health care system, and of socio-cultural and socio-economic factors. End-users, i.e. pregnant women, will not be interviewed as it would require a separate study design, and their perspective will be indirectly included through the participation of community health workers at stakeholder meetings.

For peer review only - http://bmjopen.bmj.com/site/about/guidelines.xhtml
